# Supplementary material for: ‘Communication, that is the key’: a qualitative investigation of how essential workers with COVID-19 responded to public health information
Source: BMJ Open. 2022 Jul 7;12(7):e061583. doi: 10.1136/bmjopen-2022-061583 (PMC9263375; doi:10.1136/bmjopen-2022-061583)
Supplement: Author's manuscript [file bmjopen-2022-061583.draft_revisions.pdf]

BMJ Open is committed to open peer review. As part of this commitment we make the peer review history of every article we publish publicly available.

When an article is published we post the peer reviewers' comments and the authors' responses online. We also post the versions of the paper that were used during peer review. These are the versions that the peer review comments apply to.

The versions of the paper that follow are the versions that were submitted during the peer review process. They are not the versions of record or the final published versions. They should not be cited or distributed as the published version of this manuscript.

BMJ Open is an open access journal and the full, final, typeset and author-corrected version of record of the manuscript is available on our site with no access controls, subscription charges or pay-per-view fees (http://bmjopen.bmj.com).

If you have any questions on BMJ Open's open peer review process please email info.bmjopen@bmj.com

BMJ Open

"Communication, that's the key": A qualitative investigation of how confirmed cases responded to COVID-19 information

Journal:	*BMJ Open*
Manuscript ID	bmjopen-2022-061583
Article Type:	Original research
Date Submitted by the Author:	02-Feb-2022
Complete List of Authors:	Roe, Mark; University College Dublin<br>Buggy, Conor; University College Dublin<br>Ingram, Carolyn; University College Dublin<br>Codd, Mary; University College Dublin<br>Buckley, Claire; Health Service Executive<br>Archibald, Mary; University College Dublin<br>Rachwal, Natalie; University College Dublin<br>Downey, Vicky; University College Dublin<br>Chen, Yanbing; University College Dublin<br>Sripaiboonkji, Penpatra; University College Dublin<br>Drummond, Anne; University College Dublin<br>Alvarez, Elizabeth; McMaster University, Health Research Methods, Evidence and Impact; McMaster University, Centre for Health Economics and Policy Analysis (CHEPA)<br>Perrotta, Carla; University College Dublin, School of Public Health, Physiotherapy and Sport Sciences
Keywords:	COVID-19, Public health < INFECTIOUS DISEASES, Health policy < HEALTH SERVICES ADMINISTRATION & MANAGEMENT

SCHOLARONE™
Manuscripts

I, the Submitting Author has the right to grant and does grant on behalf of all authors of the Work (as defined in the below author licence), an exclusive licence and/or a non-exclusive licence for contributions from authors who are: i) UK Crown employees; ii) where BMJ has agreed a CC-BY licence shall apply, and/or iii) in accordance with the terms applicable for US Federal Government officers or employees acting as part of their official duties; on a worldwide, perpetual, irrevocable, royalty-free basis to BMJ Publishing Group Ltd ("BMJ") its licensees and where the relevant Journal is co-owned by BMJ to the co-owners of the Journal, to publish the Work in this journal and any other BMJ products and to exploit all rights, as set out in our licence.

The Submitting Author accepts and understands that any supply made under these terms is made by BMJ to the Submitting Author unless you are acting as an employee on behalf of your employer or a postgraduate student of an affiliated institution which is paying any applicable article publishing charge ("APC") for Open Access articles. Where the Submitting Author wishes to make the Work available on an Open Access basis (and intends to pay the relevant APC), the terms of reuse of such Open Access shall be governed by a Creative Commons licence – details of these licences and which Creative Commons licence will apply to this Work are set out in our licence referred to above.

Other than as permitted in any relevant BMJ Author's Self Archiving Policies, I confirm this Work has not been accepted for publication elsewhere, is not being considered for publication elsewhere and does not duplicate material already published. I confirm all authors consent to publication of this Work and authorise the granting of this licence.

For peer review only

"Communication, that's the key": A qualitative investigation of how confirmed cases responded to COVID-19 information

Mark Roe[1], Conor Buggy[1], Carolyn Ingram[1], Mary Codd[1], Claire Buckley[2], Mary Archibald[1], Natalie Rachwal[1], Vicky Downey[1], Yanbing Chen[1], Penpatra Sripaiboonkji[1], Anne Drummond[1], Elizabeth Alvarez[3], Carla Perrotta[1]

[1] School of Public Health, Physiotherapy and Sports Science, University College Dublin, Ireland

[2] Health Service Executive, Dublin, Ireland

[3] Department of Health Research Methods, Evidence and Impact, McMaster University, Canada

Corresponding author
Mark Roe
School of Public Health, Physiotherapy, and Sports Science
Health Science Building
University College Dublin
Dublin
D04 V1W8
Ireland

Email: mark.roe@ucd.ie
Phone: +353 1 716 7777

Word count: 3,786

Tables: 4

Figures: 1

For peer review only

Patient and public involvement

Patients and/or the public were not involved in the design, or conduct, or reporting, or dissemination plans of this research.

Patient consent for publication

Not required.

Ethical approval

Ethical approval was granted by the UCD Human Research and Ethics Committee (ID: LS-E-21-85-Roe-Perrotta). Verbal informed consent was obtained from the study participants.

Provenance and peer review

Not commissioned; externally peer reviewed.

For peer review only

ABSTRACT

Objectives

To understand how essential workers with confirmed infections responded to information on COVID-19.

Design

Qualitative analysis of semi-structured interviews conducted in collaboration with the national contact tracing management programme in Ireland.

Setting

Semi-structured interviews conducted via telephone and Zoom Meetings.

Participants

18 people in Ireland with laboratory confirmed SARS-CoV-2 infections using real-time polymerase chain reaction testing of oropharyngeal and nasopharyngeal swabs. All individuals were identified as part of workplace outbreaks defined as ≥2 individuals with epidemiologically linked infections.

Results

A total of four high-order themes were identified: (1) Accessing essential information early, (2) responses to emerging “infodemic”, (3) barriers to ongoing engagement, and (4) communication strategies. Fourteen lower-order, or subthemes, were identified and agreed upon by the researchers.

Conclusions

Our findings provide insights into how people infected with COVID-19 sought and processed related health information throughout the pandemic. We describe strategies used to navigate excessive and incomplete information and how perceptions of information providers evolves overtime. These results can inform future communication strategies on COVID-19.

For peer review only

STRENGTHS AND LIMITATIONS

- The qualitative design enabled the collection of data on the experiences and perceptions of people who had laboratory confirmed COVID-19 infections.
- Throughout the study the researchers used several best-practice strategies to complete thematic analysis of data from semi-structured interviews. Additionally, given the lack of prior qualitative research on this topic and cohort, we used inductive reasoning approach which enables us to avoid assumptions of prior research or frameworks.
- Participants were selected from sub-group of the population known as 'essential workers' that could not avail of 'remote working' throughout the pandemic. Thus, their views are critical to informing public health policies that cater for cohorts at increased risk of infection.
- A potential limitation of this study is that our recruitment strategy had a low response rate perhaps indicating a low willingness in this cohort to engage with COVID-19 research or initiatives associated with official public health organisations. This may bias our sample and could be addressed through future contact tracing strategies.

For peer review only

BACKGROUND

Sharing public health information is a crucial step in creating awareness of threats and protective strategies.[1] The COVID-19 pandemic is unique in two ways. First, it is the first pandemic where digital technologies and social media platforms are used to share information to keep people safe.[2] Second, although little information was available on the novel SARS-CoV-2 pathogen in January 2020, an 'infodemic' had emerged within four months, prompting global responses such as the World Health Organization's Information Network for Epidemics (EPI-WIN).[3]

While acknowledging of wide-spread access to information on COVID-19, little is known about how people receive and process such content. This is especially true for groups at a higher risk of infection who are associated with super-spreading events (SSEs) which have a greater influence on the trajectory of epidemics.[4]

Prior research on COVID-19 information has explored the public's perceptions of communication strategies, and their ability to correctly answer questions on COVID-19 epidemiology.[5, 6] These offer little insight into how people who were most at risk of infection (e.g. high risk workers) make sense of information on COVID-19 or how they believe communication strategies could be improved in the future.

Using semi-structured interviews, understand how essential workers with confirmed infections responded to information on COVID-19.

METHODS

This study represents a sub-analysis of a preregistered study to investigate the behaviours and contextual factors associated with COVID-19 outbreaks and SSEs (OSF: https://osf.io/aeg74).

Patient and public involvement

There was no patient or public involvement in setting the research agenda.

Data collection methods

Semi-structured interviews were conducted using a password-protected Zoom account via a secure login. Only the researcher and invited participant were admitted

For peer review only

to the call. A transcript of the audio recording was generated and corrected following a playback of the original audio file, enabling the researchers to remove any identifiable information. Each participants was assigned a unique identification number (PID). After this process, recordings were deleted from all sources. Four researchers completed the interviews following a series of pilot interviews with fellow team members (NR = 8; MR = 6; MA = 2; CI = 2). All interviews were conducted from 22nd July 2021 to 7th Aug 2021.

For context, as of 22nd July 2021, Ireland reported 289,139 laboratory confirmed infections since the first case on 29th February 2020, equating to a population prevalence of 5.8%.[7] The median age of national cases was 22. Healthcare workers accounted for 10.3%. A total of 15,134 hospital admissions and 5,026 were reported. Approximately, 63.8% and 53.0% of the population had been partially or fully vaccinated against COVID-19. In the 14-days prior to data collection, Ireland confirmed 13,065 infections (prevalence = 0.3%) with 29.5% of cases associated with community transmission.[8] The median age was 23. A similar infection rate was reported until 13th Oct 2021. Ireland's 7-day test positivity rate was 5.9-7.9% throughout the study period.[7]

We developed an interview guide to explore three areas: behaviours and contextual factors leading to COVID-19 outbreaks, responses to minimise transmission, and lessons for future responses. Within this we asked specific questions on information and public health messaging and the role of the media in minimising transmission and sustaining safe behaviours. Prompt questions were asked by the researchers to illicit more detailed information on these topics.

Study participants

We recruited 18 people in the Republic of Ireland who had laboratory-confirmed SARS-CoV-2 infections using real-time polymerase chain reaction testing of oropharyngeal and nasopharyngeal swabs. All individuals were identified as part of suspected workplace outbreaks where ≥2 individuals had epidemiologically linked infections. We targeted this group given their first-hand experience of (1) COVID-19 outbreaks, and (2) responses to minimise transmission. Thus, inclusion criteria

For peer review only

included age ≥18 y and a laboratory confirmed infection likely linked to a workplace outbreak.

Potential participants (n=167) were identified by the partner Health Service Executive (HSE) Contact Tracing Centre (CTC) as fulfilling the inclusion criteria, and during routine surveillance activities, were invited to receive a call from the research team. Overall, 104 individuals were contactable by the HSE CTC (62.3%). From this cohort, 64 (61.5%) refused to be contacted by the research team while 40 (38.5%) gave consent. We were able to contact and complete interviews with 18 people from this sample, representing 10.8% of potential participants originally identified by the HSE CTC.

Seven females (38.9%) and 11 males (61.1%) were interviewed. The median age was 35 y (min = 18; max = 57). Sectors represented included food, construction, laundry, retail, energy, healthcare, professional services, public services, and emergency services.

Data analysis

We used a thematic analysis approach to support a structured exploration of patterns in perceived behaviours and contextual factors associated with COVID-19 outbreaks experienced by the participants.

The coders (MR x 10, VD x 8) familiarised themselves with the transcripts prior to initiating coding. Thematic analysis was conducted in accordance with the six phases of trustworthy thematic analysis as outlined by Nowell et al. (2017) (i.e. familiarisation with the data; generating initial codes; searching for themes; reviewing themes; defining and naming themes; producing the report).[9] Qualitative analysis software (NVivo V12) was used to transform raw data into multiple codes using open and axial coding processes. A final of 35 codes were computed.

We considered saturation was met when two conditions were realised: (1) agreement between researchers that no further transcript codes could be generated, and (2) no further information, deemed relevant to the stated research questions, could be obtained from the transcripts.[10] Discrepancies were resolved through discussion until a consensus was reached. Reflective discussions were conducted (MR, VD, CI)

For peer review only

throughout the study to review the rationale for selecting codes, themes, and quotes.[11] Credibility strategies used throughout analysis included (1) critical friend approach and (2) cross-checking of themes identified in prior focus group studies. The team has a diverse range of background (e.g. medicine, public health, epidemiology, risk management, occupational health, psychology) that facilitated a robust review of identified themes. Results are reported in line with SRQR reporting guidelines (Supplementary file 1).[12]

RESULTS

A total of four high-order themes were identified: (1) Accessing essential information early, (2) responses to emerging infodemic, (3) barriers to ongoing engagement, and (4) communication strategies. Fourteen lower-order, or subthemes, were identified and agreed upon by the researchers (Figure 1).

Theme 1: Accessing essential information early

Sub-theme 1.1: Clarity on what to know and do to prevent infection

Participants expressed the view that since COVID-19 was first confirmed in Ireland on 29th February 2020, public health information was almost unavoidable given the extent of coverage on news programmes and social media. Most participants accessed information via national television news shows which included live broadcasts of public health press briefings and newspapers as well as websites of the HSE and World Health Organization. Additionally, every participant reported having access to information on COVID-19 in their workplace. Workplace information provided clarity on what staff could expect if workers developed symptoms of COVID-19. Interestingly, it appears that prior to their infection, participants largely tended to only seek generic information on COVID-19 that was deemed necessary for functional tasks (table 1).

Sub-theme 1.2: Personal scenarios triggering in-depth information seeking

Participants referred to triggers that initiated searches to answer specific queries related to COVID-19 that arose from unique personal events (table 1). This was largely indicative of personal concerns related to changes in their own health status, or that of an acquaintance or family member, leading to a reappraisal of the threat posed

For peer review only

Table 1 – Illustrative quotes supporting theme one 'accessing essential information early'

Sub-Theme	Supporting Quotes	Participant ID
1.1 Clarity on what to know and do to prevent infection	You were kind of just told what the symptoms were, and you were educated on what symptoms to look out for and if you have any of these symptoms to report them and to isolate at home.	PID 12: Nurse, female, 30 y
	Everyone was already pretty much informed of what would happen if there was a scenario where someone gets sick.	PID 17: Meat plant worker, male, 20 y
	The procedures were rolled out nationally. There was no issue with dissemination of information. The quality of information coming at a national level, it was good.	PID 20: Emergency services worker, male, 35 y
	There's a lot of access [to information] in every part of the company. [It's been] very very good through the COVID period so I give out credit for that.	PID 7: Food factory worker, female, 35 y
	I got the information I needed, I never looked for any more information that I didn't need.	PID 12: Nurse, female, 30 y
1.2 Personal scenarios triggering in-depth information seeking	It's [not] until either they get COVID or they see someone that they know get COVID badly, once that happens then they'll realize what COVID is, and that's the sad reality is that there's a lot of people who won't take this thing seriously until they see somebody that they know with COVID.	PID 1: Meat plant worker, male, 18 y
	When I got the headache I obviously was looking up to see was this part of the symptoms. Is it actually COVID or is it just this cough I had before Christmas? So I definitely was seeking out symptom information, and I read, I don't know it was like a dissertation or a there was a paper written on this 72 hour headache that wouldn't react to any medication or anything so that kind of rang true.	PID 16: Office worker in professional services, female, 27 y
1.3 Infection triggering clinical information seeking	I won't really be looking for information unless I like start showing symptoms and I start to feel very unwell then I'll start looking at it going like, right, what is it, how can I help myself with this, what can I do.	PID 12: Nurse, female, 30 y
	[At the time] I was just kind of reading through different information about symptoms and how long they last and different things that can happen.	PID 16: Office worker in professional services, female, 27 y

For peer review only

COVID-19. More in-depth information was sought from specific Google searches, YouTube videos, and formal research papers. It appears these intentional information seeking activities served the specific purpose of helping the participants make sense of their personal scenarios.

Sub-theme 1.3: Infection triggering clinical information seeking

We also noticed that even following confirmation of a positive test, participants only sought information when COVID-19 began to impact their health (table 1). A clear distinction was noted between this and sub-theme 1.2, as information seeking occurred after a specific event (i.e. following confirmed infection) and refers to the specific domain of case management (i.e. what are the potential clinical outcomes, and how what actions can support better outcomes). Participants expressed that these searches for specific clinical information allowed them to prepare for what might be expected to occur throughout their period of self-isolation.

Theme 2: Response to emerging infodemic

Sub-theme 2.1: Frustration with misleading headlines

We repeatedly found references to perceived over-reporting on COVID-19 by the media (table 2). Whilst participants mentioned that although the sustained media coverage was at times fear-provoking, it also played a useful role in keeping the public informed of the latest COVID-19 trajectory. Participants reported being disheartened by the manner of reporting, as opposed to the constant publication of media content related to COVID-19. Similarly, participants also alluded to their distaste for some marketing tactics, deployed by some media outlets to increase online traffic, which had the potential to misinform the public.

Sub-theme 2.2: Desire for factual information on local area events

Our analysis identified that participants valued information related to their local geographical area, going beyond the reporting of mere confirmed case numbers to reveal details of infection sources and clinical outcomes (table 2). This was seen as beneficial to raising awareness of the threat in the area in attempts to bolster adherence to infection control measures.

For peer review only

Table 2 – Illustrative quotes supporting theme two 'response to emerging infodemic'

Sub-Theme	Supporting Quotes	Participant ID
2.1 Frustration with misleading headlines	I feel like the cases are kind of annoying hearing about [them] every day, but I feel like it is good to know… I think they just report on it too often kind of scaring people like as in they kind of use headlines to scare people, when you read the actual information it's very misleading.	PID 14: Factory worker, female 22 y
	Give proper factual information instead of kind of click bait headlines on everything.	PID 14: Factory worker, female 22 y
2.2 Desire for factual information on local area events	There should be maybe more information on it like how many people are like in hospital in each area and like where the cases are explicitly coming from. [I] think it'd be more beneficial than maybe just hearing the cases every day, you kind of stop listening and stop caring, there's like 1,000 cases when like 4 million people like it doesn't, but if you kind of read it and know people are near you it might make people pay more attention and stick to the rules and keep safe	PID 14: Factory worker, female 22 y
	I think the government needs to be more transparent on things and provide more accurate information because at the time when this whole pandemic was rampant I feel like the government was just overloading people with so much information that you didn't even know which one is right which one is wrong. You could be saying 'oh that's what I heard is' and then another person could be saying 'oh no, this is what I heard is' and like you know it's all a bunch of everything. All the information is all jumbled up and the truth is somewhere in the middle.	PID 17: Meat plant worker, male, 20 y
2.3 Understanding what to focus on	It's just knowing what this virus is and knowing how it's going to affect your body and being able to prepare yourself for when it's going to affect your body	PID 1: Meat plant worker, male, 18 y
	I was just reading more about it and what it actually does and how or what I'm supposed to take or do to make my immune system stronger, so those were the things I was checking	PID 3: Laundry worker, male 28 y
	I guess the main thing is have information readily available for everybody just to keep on track and keep updated on what the most recent guidelines are.	PID 12: Nurse, female, 30 y

For peer review only

Sub-theme 2.3: Understanding what to focus on

Despite repeated references to excessive amounts of information on COVID-19 in the media and online, participants appear to have coped by considering the relevance of the content for understanding the nature of the virus, and how it could inform their future actions (table 2).

Theme 3: Barriers to ongoing engagement

Sub-theme 3.1: Experiences leading to a low risk perception

We noted that several participants, having reflected on how the congruency between their personal experiences throughout the pandemic and those reported by the media, expressed a current belief that COVID-19 was not as dangerous as reported in the media (table 3). These participants believed that they correctly understood the content of prior media reports, yet witnessed, or heard about, contradictory COVID-19 outcomes prior to adopting their alternative beliefs. A source of evidence for personal experiences leading to a lower risk perception appears to be perceived wrong information that was reported yet never acknowledged or corrected. We noted that such participants were eager to emphasise that they were not denying the existence of COVID-19.

Of 18 participants, we encountered only one that alluded to conspiracy theories. Thus, we draw a clear distinction between the emergence of alternative hypothesises, at odds with the narratives reported by official authorities or media outlets, and conspiracy theories citing malicious motives by the official organisations.

Sub-theme 3.2: Zoning out from formal communications

Participants referred to a reduced ability to continually engage with media and public health messages over time, resulting in unintended outcomes such as when some people become “accustomed to hearing about it and stop caring”. Formal communications strategies (i.e. content released by national health authorities such as press briefings) on COVID-19 in Ireland were first implemented in February 2020. We noted the belief that the public developed a reduced willingness, or capacity, to engage in this form of messaging as the pandemic progressed (table 3). Instead,

For peer review only

participants mentioned the role of 'informal advertisements', associated with the placing of public health messages during the start and end of popular programs and delivered by the host.

Sub-theme 3.3: Forming inflexible opinions

Participants recognised the role of social networks, particularly those online, in shaping peoples' interpretation of information related to COVID-19. Specifically, we noted the perception that online social networks were effective at facilitating a process whereby users arrived at an interpretation of COVID-19 information that was likely to be anti-establishment (table 3).

Sub-theme 3.4: Turning against the government

Participants spoke of the consequences of prior measures taken to reduce transmission of COVID-19. In particular, national lockdowns, known locally as 'Level Five restrictions' were identified as being a source of anger amongst the public. We found that participants were unlikely to dissociate government officials from senior members of Ireland's National Public Health Emergency Team. Additionally, there was a sentiment that the public's negative reaction to previous COVID-19 responses would lead some people to adopt a negative attitude of future messages regardless of the content (table 3).

Theme 4: Communication strategies

Sub-theme 4.1: Personal motives for paying attention

Despite referring to excessive information and media coverage, all participants appeared to value ongoing communication. Similar to our findings in sub-theme 1.2 (i.e. personal scenarios triggering in-depth information seeking), participants expressed a need for future communications to be anchored to a personal value proposition, such as protecting loved ones (table 4).

Sub-theme 4.2: Dealing with misleading information

All participants used social media yet were unclear about specific measures that could be taken to reduce the misinformation on digital platforms (table 4).

For peer review only

Table 3 – Illustrative quotes supporting theme three ‘barriers to ongoing engagement’

Sub-Theme	Supporting Quotes	Participant ID
3.1 Experiences leading to a low risk perception	I believe soon it’s coming, another lockdown. Soon, very soon I believe, and I think that you know that already. There are so many cases that’s why. Because I said like I don’t know how [they're] can be so many cases. But we never... heard, like we saw on the news there are few cases like 900 or something like that a day... but we didn’t hear like somebody had for real COVID.	PID 22: Meat plant worker, female, unknown age
	It’s not like we don’t believe about COVID, alright it’s a virus I understand, but it’s not as worse [as] they actually say… How can you believe everything they say on the news and everything when people every day see different?	PID 21: Construction worker, male, unknown age
3.2 Zoning out from formal communications	So the only information I really looked for was [when symptomatic], I was talking to my boss at the time so he was the one who was keeping me informed with what I should be doing at all, so uh he told me to stay off. I think it was 2 days and then if I still had symptoms or if symptoms [hadn't] got better I was to get a test at the GP.	PID 17: Meat plant worker, male, 20 y
	I feel like some people when they kind of read the news like what will make them be more careful, but sometimes it’s the opposite. Like if people are hearing it every day they just kind of just become accustomed to hearing about it and stop caring.	PID 13: Nurse, female, 27 y
	You always hear [TV host] when he’s on [Ireland’s most popular entertainment show] wrapping it up saying “everyone make sure to wear your masks and wash your hands”, so if it was kind of embedded into more of an informal advertisement. I think it could be responded to better or even like on the radio channels just that it's nearly embedded subconsciously into people. But I think the formality on how they’re doing it now, it’s just lost the interest of people. People are just zoned out they don’t care anymore about it.	PID 13: Nurse, female, 27 y
3.3 Forming inflexible opinions	People are very headstrong. They'll keep this thought that they have of whatever their opinion on COVID is because, like, they'll have one or two people agree with them on Facebook or something, and they’ll think alright well what I’m saying is correct.	PID 1: Meat plant worker, male, 18 y
	Some people like they read too much online on Facebook, on whatever like Instagram or whatever. And then [get] sucked into it and then they end up believing it, and then it just keeps going on and on until they're sitting there telling people that COVID isn’t real or that the vaccine is trying to control them. But, like, it's just that some people think that anything they're told shouldn't be taken at face value. Some people find it hard that when the government says something, that’s the truth, some people do find that very hard.	PID 1: Meat plant worker, male, 18 y
	The main thing is, especially with the measures that we had in place when we were in Level Five, I think those were very, they were very harsh. I understand. Yet it did work. But it didn't really. Like, there were a lot of people that turned against the government.	PID 1: Meat plant worker, male, 18 y
	I think to be honest with ya a lot of people have a negative response to the government and most of the directors I’ve heard from the government. I know a lot of people don’t like [Ireland’s Chief Medical Officer]. I don’t mind him, but a lot of people don’t like him, and if they say something they’re [of] the attitude that they're not just going to take it on board, they’re just going to be disrespectful to him and it’s just going to ignored from the get go.	PID 13: Nurse, female, 27 y

For peer review only

Table 4 – Illustrative quotes supporting theme four 'communication strategies'

Sub-Theme	Supporting Quotes	Participant ID
4.1 Personal motives for paying attention	Communication that's the key to explain to them, to explain things to them because if you love someone, your parent, if your parent is vulnerable, you need to protect them, we protect each other… if I protect myself, I protect also my family… If we pay attention [to] the information, we can protect each other.	PID 4: Laundry worker, male, 57 y
	The information being disseminated is continued and is of good quality but that depends on the individual person, whether they decide to look at that information or read it. There was no onus on anybody to read it and if they didn't it wouldn't make a whole lot of difference to be honest with ya.	PID 20: Emergency services worker, male, 35 y
	I think some people, they should get more information when we know [that COVID-19] is really happening in the world. So me and my daughter and my family we got [the] most information we could. But all the time we leave we put mask on, alcohol gel we have in handbag, all the time using sanitizer to clean the doors and stairs and everything. But when I start work, I see some people they doesn't care so much, you know.	PID 7: Food processing plant general manager, female, 35 y
	I mean one key piece of information that I'd say is make sure people know that in the case of testing positive they won't go without pay. Because like I can see that causing a lot of unrest with people because you know it's like I said, a lot of people have families to feed you know and people rely on them for the money to get by. If people knew that and it's like 'oh I'll be taken care of if this happens' you know, they'll be a lot more willing to cooperate with the normal guidelines.	PID 17: Meat plant worker, male, 20 y
4.2 Dealing with misleading information	Maybe if there was something in place to kind of stop all the false news and kind of stuff that's spread online.	PID 14: Factory worker, female 22 y
	[When tested positive] the HSE [national health service] send me email for ten days about what I do [and to ask] how I feel.	PID 9: Meatplant worker, male, 56 y
4.3 Strategies to maintain credibility	All the guys are doing everything that [they] can do because there's so much more information about this... Because everything we know we get it from HSE [Health Service Executive], we get it from news, we get it from TV. Like everything, we get it from information from the health organisations giving to us.	PID 7: Food processing plant general operations, female, 35 y
	It's just about reinforcing the information, the guidelines. It's a bit like any other training that we do in healthcare when it comes to basic life support or infection control or anything like that. We do annual updates of that every year and that is the simple purpose to keep the practice up, to keep your skills up and to keep it on the forefront of your mind so that you don't kinda forget and and let standards slip. I think that's really the best way really to go forward, is to have support if you have any questions and have that support readily available but as well as that just having the information there and having regular updates and what's best to do.	PID 12: Nurse, female, 30 y
	[When infected] I had access from the [contact tracing caller] to all the information. I feel like they had all the information.	PID 8: Meatplant worker, male, 41 y

For peer review only

4.4 Desire for vivid warnings and sharing personal stories	They should have a picture of what COVID does to your body and they'll help people realize because, like, on the smoking packets, it does help people realise and they say on them like the dangers of smoking. It shows the harsh reality of it, and that's what people need to realise is the harsh reality.	PID 1: Meat plant worker, male, 18 y
	From a public point of view, all I can say is again information. Just that information is put out there readily for everyone. That it's clear, it's concise, it's honest, and it's blunt with regards to the impact that it will have and what's expected.	PID 12: Nurse, female, 30 y
	Like I got infected. I could tell them, 'this is my story and yes it [is] real. You should believe this'.	PID 3: Laundry worker, male, 28 y
	Thank you for calling and thank you for letting me explain my experience because it's very hard when you are alone. Like I'm single mother, just me and my daughter, so if something happen with me I was scared with who was going to look after my baby and all this things. But at same time [being infected] make me more strong, make me look ahead and say listen 'this is going to pass, this is not going to affect you forever, this is just a moment, uh, so keep calm'.	PID 7: Food processing plant general operations, female, 35 y

For peer review only

Sub-theme 4.3: Strategies to maintain credibility

Our analysis indicates that to avoid misinformation, participants adopted a strategy of seeking information from several recognised health authorities, rather than a sole source, before cross-checking the content with media reports (table 4).

Sub-theme 4.4: Desire for vivid warnings and sharing personal stories

When considering lessons for future strategies to improve adherence to public health measures, participants again referred to personal scenarios. Specifically, strategies used to respond to promote awareness of other public health threats, such as smoking, were mentioned by participants (table 4).

DISCUSSION

Our study aimed to understand how people sought and processed information on COVID-19. Analysis of semi-structured interviews with 18 participants identified four themes: (1) accessing essential information early, (2) response to emerging infodemic, (3) barriers to ongoing engagement, and (4) communication strategies. No hypothesis was tested. Instead, we used inductive reasoning to explore patterns in COVID-19 information usage, identifying several findings that can inform public health information strategies.

The precursor for seeking information is a perceived need to understand (i.e. describe what is) and respond (i.e. prescribe what should be done).[13] These needs evolve overtime, are directed by personal experiences, and determine the perceived importance of seeking the best possible information or making correct decisions.[14]

Given the extent of coverage in the media, information on COVID-19 was practically unavoidable in 2020. Early information on COVID-19 appears to have had the effect of focusing attention on symptoms and how suspected cases should be managed. These early 'fundamental truths', even amid incomplete information, were simple to process and capable of informing public behaviours.[15] This is important as most of the public in the region of study has had no prior experience of pandemic pathogens.

We identified that most participants obtained information from official organisations such as national and international health organisations, indicating that people trust

For peer review only

these sources. In some instances, failure to share the best available information led people to seek info from misleading sources. Disengagement is happening and can be problematic. Yet as it happens, we see examples of lived experiences contributing to healthy behaviours. Thus, a solution might be to facilitate exchanges between producers of information and the general population to (1) ensure that needs and population expertise are being heard and (2) encourage engagement by building trust through dialogue and providing ongoing explanation.

Prior studies provide evidence that the perceived risk of infection depends on the proportion of neighbours that are ill, meaning that amid a lack of official communication on local events, people turn to personal networks to gain information on their neighbourhood.[16] In the era of digital networks, this inevitably funnels people towards social media platform, increasing exposure to misinformation and polarising opinions.[17]

We note calls for social media companies to regulate information deemed inaccurate, false, or malicious.[18] However, that is unlikely to satisfy information needs as people will most likely default to word of mouth.[19] For instance, Lupton and Lewis (2021) noted that the public use informal communications with friends and families, local and internationally, to keep up to date with emerging information.[20]

Whilst the role of clickbait headlines and social media is undoubtedly playing a role in spreading misinformation, so too is the inability of public health authorities to collect and disseminate information to people on live outbreaks in their communities. Surveillance capacities are a key component of the International Health Regulations for preparing and responding to infectious disease threats.[21] Tools such as community outbreak alerts might be particularly useful, especially as attention to specific diseases may decline after surges of infection when relatively low national figures are communicated.[16]

Although tools such as community outbreak alerts are common in many jurisdictions, Irish health authorities have not used them citing concerns related to data privacy, however, guidance from the national Data Protection Commissioner on 6th Mar 2020 states that "Article 9(2)(i) of [General Data Protection Regulations] GDPR and Section 53 of the Data Protection Act 2018 will permit the processing of personal data,

For peer review only

including health data, once suitable safeguards are implemented. Such safeguards may include limitation on access to the data, strict time limits for erasure, and other measures such as adequate staff training to protect the data protection rights of individuals".[22]

It is foreseeable that people receiving information at odds with their personal experiences creates doubts in credibility of sender. Our results imply that overtime, participants adopted a cautious attitude or scepticism towards information shared by official and government organisations, indicating a degree of frustration and mistrust.[23]

Gaps in information lead to more reliance on experiential knowledge, a recognised useful resource when responding to uncertainty (e.g. learning from community leaders or professional mentors) especially if derived in settings that are difficult for outsiders to grasp.[24, 25] This is likely attributable to concerns about COVID-19, and the response to it, 'bubbling up' slowly overtime as the pandemic progresses.[26] Factors contributing to these changes in personal risk perceptions included delays in collecting and reporting information, the presence of random noise in that information, and the shared belief in social networks that the posed risk is reducing over time.[27] Hence, we observed an interplay between people making an effort to stay informed whilst simultaneously withdrawing disengaging from previously trusted sources, leading to emergence of more settled, and potentially alternative, beliefs running counter to official interpretations.[23] We believe this is also evident in our ability to only reach 10.8% of our identified sample.

Adopting beliefs by processing imperfect information through social interactions indicates the use of well-documented heuristic principles, mainly the representativeness heuristic (i.e. comparing risk situations with those that seem similar), the availability heuristic (i.e. assessing risk with information most easily accessed), and the anchoring heuristic (i.e. risk estimate based on previous event).[14, 27] Whilst this may seem undesirable to evidence-based domains, heuristics and risk management principles continue to form the basis of many effective COVID-19 measures that are defendable even in the absence of scientific evidence.[28, 29]

For peer review only

Additionally, the true risk of COVID-19 events has been difficult to accurately assess and provide information on. For example, as evidenced by large differences between reported case numbers and seroprevalence studies.[30] Overtime, as dissatisfaction manifests and trust in the information sender is eroded, it is unlikely that responses such fact-checking will deliver an immediate change in perception or behaviour.[31] In line with the action-based model of cognitive-dissonance processes, the sensation leading to a "zoning out", signalling a distancing from pre-trusted sources, as inconsistencies in information and cognition must be resolved through alternatives means for the person to take logical course of action.[32]

Hence, it is oversimplistic to label 'zoning out' as a symptom of a catch-all term like pandemic fatigue (i.e. a perceived exhaustion of cognitive and emotional capacities to sustain the effort required to positively respond to outbreaks), given that it is influenced by a complex two-way sender-receiver process of detecting, disseminating, and deciphering key data, including outcomes of policies adopted by public health officials and government. Instead, it is perhaps more accurate to consider 'zoning out' as a stage in response to incomplete information from formal releases from state agencies that leads people to move away from previously reliable sources to seek additional information.

Participants referenced previous public health campaigns that involved graphic health warnings on cigarette packaging. Research has shown that the public have a preference for larger, pictorial, and loss-framed warning labels given that these are more likely to attract attention and further thought or information seeking on health risks.[33] Such communication strategies have been shown to be particularly effective at generating motivation to change behaviour in cohorts aged 13-30 y; a group more likely to online social media.[34, 35]

We also note similarities between our findings on communication strategies and those from a thematic analysis of social media posts linked to a video-based quit smoking campaign that exposed the public to comments deemed both oppositional (e.g. role of government, bigger perceived threats, scientific scepticism) and supportive (e.g. personal stories, support for change, reactions to ad content).[36] Critics of graphic health warnings often believe that these strategies are emotional and not factual,

For peer review only

however, evidence suggests that there is little difference in emotional response to information presented in graphic or factual text form, suggesting a false dichotomy.[35]

Whilst sustained communication can overwhelm, it is also a valued strategy to alleviate anxieties and uncertainty. For messages to provide clarity they must recognise both the change in information and misinformation.[20] This is because the public recognise that COVID-19 misinformation is common and expect scientists and governments to provide accurate information.[20]

Given the ever-evolving nature of infectious pathogens, the rapid detection and dissemination of health threats are heavily reliant on IT infrastructure for two-way communication between health providers and the public.[21, 23]

We also note references to sharing stories by participants. Like Bennett et al., we believe that future communication strategies could learn from personal experiences of people infected with COVID-19 if given “an outlet [to hear their] uncensored stories”.[37]

STRENGTHS AND LIMITATIONS

Our novel study design involved a partnership with the national contact tracing management programme. The qualitative design enabled the collection of data on the experiences and perceptions of people who had laboratory confirmed COVID-19 infections. Throughout the study the researchers used several best-practice strategies to complete thematic analysis of data from semi-structured interviews. Additionally, given the lack of prior qualitative research on this topic and cohort, we used inductive reasoning approach which enables us to avoid assumptions of prior research or frameworks. Participants were selected from sub-group of the population known as ‘essential workers’ that could not avail of ‘remote working’ throughout the pandemic. Thus, their views are critical to informing public health policies that cater for cohorts at increased risk of infection.

A potential limitation of this study is that our recruitment strategy had a low response rate perhaps indicating a low willingness in this cohort to engage with COVID-19 research or initiatives associated with official public health organisations. This may bias our sample and could be addressed through future contact tracing strategies.

For peer review only

Contributorship Statement

All authors conceptualised and designed the study, and reviewed and revised the manuscript. MR, CI, MA, NR, CP, MC, and CB designed the data collection instruments and recruited participants. MR, CI, MA, and NR conducted qualitative interviews. MR, VD, and CI analysed qualitative data. MR and CI drafted the initial manuscript. All authors approved the final manuscript as submitted and agree to be accountable for all aspects of the work.

Competing interests

None declared.

Data availability statement

Data are available on reasonable request. Raw transcripts of the data analysed in the study are available from the lead author.

Funding

This work was supported by Science Foundation Ireland (SFI) through the COVID-19 Rapid Response Research and Innovation Programme, grant number 20/COV/8539.

For peer review only

References

1. Mheidly N, Fares J. Leveraging media and health communication strategies to overcome the COVID-19 infodemic. J Public Health Policy. 2020;41(4):410-420. doi: 10.1057/s41271-020-00247-w.

2. Fagherazzi G, Goetzinger C, Rashid MA, Aguayo GA, Huiart L. Digital Health Strategies to Fight COVID-19 Worldwide: Challenges, Recommendations, and a Call for Papers. J Med Internet Res. 2020;22(6):e19284. doi: 10.2196/19284.

3. World Health Organization: About EPI-WIN. Accessed at: https://www.who.int/teams/risk-communication/about-epi-win

4. Broomell S, Kane P. Perceiving a pandemic: Global–local incompatibility and COVID-19 superspreading events. Decision, 2021;8(4), 227–236. https://doi.org/10.1037/dec0000155

5. Malecki KMC, Keating JA, Safdar N. Crisis Communication and Public Perception of COVID-19 Risk in the Era of Social Media. Clin Infect Dis. 2021;72(4):697-702. doi:10.1093/cid/ciaa758

6. Jaber RM, Mafrachi B, Al-Ani A, Shkara M. Awareness and perception of COVID-19 among the general population: A Middle Eastern survey. PLoS One. 2021;16(4):e0250461. doi:10.1371/journal.pone.0250461

7. Government of Ireland and Ordnance Survey Ireland: Ireland's COVID-19 Data Hub. Accessed at: https://covid-19.geohive.ie/search

8. Health Surveillance Protection Centre: Epidemiology of COVID-19 in Ireland: HPSC interim 14 day report on cases uploaded to the COVID Care Tracker. Accessed at: https://www.hpsc.ie/a-z/respiratory/coronavirus/novelcoronavirus/surveillance/covid-1914-dayepidemiologyreports/2021/july2021/COVID-19_Interim%2014%20day%20report_INTERIM_20210722_WEBSITE.pdf

9. Nowell LS, Norris JM, White DE, Moules NJ. (2017). Thematic Analysis: Striving to Meet the Trustworthiness Criteria. International Journal of Qualitative Methods. https://doi.org/10.1177/1609406917733847

10. Fusch PI, Ness LR. Are We There Yet? Data Saturation in Qualitative Research. The Qualitative Report 2015;20(9):1408-1416. https://doi.org/10.46743/2160-3715/2015.2281

11. Ortlipp M. Keeping and using reflective journals in the qualitative research process. Qual Rep 2008;13:695–705.

12. O'Brien BC, Harris IB, Beckman TJ, Reed DA, Cook DA. Standards for reporting qualitative research: a synthesis of recommendations. Acad Med. 2014;89(9):1245-1251.

13. Kim HK, Ahn J, Atkinson L, Kahlor LA. Effects of COVID-19 Misinformation on Information Seeking, Avoidance, and Processing: A Multicountry Comparative Study. Sci Commun. 2020;42(5):586-615. doi:10.1177/1075547020959670

For peer review only

14. Pelletan J. Risk perception with imperfect information and social interactions: Understanding group polarization. Bulletin of Economic Research 2021;73(4):688-703.

15. Pettersson R. Information Design–Principles and Guidelines, Journal of Visual Literacy, 2010;29:2, 167-182, DOI: 10.1080/23796529.2010.11674679.

16. Bagnoli F, Liò P, Sguanci L. Risk perception in epidemic modeling. Phys Rev E Stat Nonlin Soft Matter Phys. 2007 Dec;76(6 Pt 1):061904. doi: 10.1103/PhysRevE.76.061904.

17. Kim J-N, Gil de Zúñiga H. Pseudo-Information, Media, Publics, and the Failing Marketplace of Ideas: Theory. *American Behavioral Scientist*. 2021;65(2):163-179. doi:10.1177/0002764220950606

18. Wardle C, Singerman E. Too little, too late: social media companies' failure to tackle vaccine misinformation poses a real threat. BMJ 2021; 372 :n26 doi:10.1136/bmj.n26

19. Yasir A, Hu X, Ahmad M, Rauf A, Shi J, Ali Nasir S. Modeling Impact of Word of Mouth and E-Government on Online Social Presence during COVID-19 Outbreak: A Multi-Mediation Approach. International Journal of Environmental Research and Public Health. 2020; 17(8):2954. https://doi.org/10.3390/ijerph17082954

20. Lupton, D., Lewis, S. Learning about COVID-19: a qualitative interview study of Australians' use of information sources. BMC Public Health 21, 662 (2021). https://doi.org/10.1186/s12889-021-10743-7

21. Kandel N, Chungong S, Omaar A, Xing J. Health security capacities in the context of COVID-19 outbreak: an analysis of International Health Regulations annual report data from 182 countries. Lancet. 2020;395(10229):1047-1053. doi: 10.1016/S0140-6736(20)30553-5.

22. Data Protection Commission: Data Protection and COVID-19. Accessed at: https://www.dataprotection.ie/en/dpc-guidance/blogs/data-protection-and-covid-19

23. Jennings W, Stoker G, Valgarðsson V, Devine D, Gaskell J. How trust, mistrust and distrust shape the governance of the COVID-19 crisis, Journal of European Public Policy, 2021;28:8, 1174-1196, DOI: 10.1080/13501763.2021.1942151

24. Baillergeau E, Duyvendak J. Experiential knowledge as a resource for coping with uncertainty: evidence and examples from the Netherlands, Health, Risk & Society, 2016;18:7-8, 407-426, DOI: 10.1080/13698575.2016.1269878

25. Griffin RJ, Dunwoody S, Neuwirth K. Proposed model of the relationship of risk information seeking and processing to the development of preventive behaviors. Environ Res. 1999 Feb;80(2 Pt 2):S230-S245. doi: 10.1006/enrs.1998.3940.

26. Loewenstein G, Mather J. Dynamic Processes in Risk Perception. Journal of Risk and Uncertainty 1990;3(2):155–175.

27. Tversky A, Kahneman D. Judgment under Uncertainty: Heuristics and Biases. Science. 1974;185(4157):1124–1131.

For peer review only

28. Taleb NN, Bar-Yam Y, Cirillo P. On single point forecasts for fat-tailed variables. Int J Forecast. 2020;10. doi:10.1016/j.ijforecast.2020.08.008

29. Ingram C, Downey V, Roe M, et al. COVID-19 Prevention and Control Measures in Workplace Settings: A Rapid Review and Meta-Analysis. Int J Environ Res Public Health. 2021;18(15):7847. doi:10.3390/ijerph18157847

30. Rostami A, Sepidarkish M, Leeflang MMG, Riahi SM, Nourollahpour Shiadeh M, Esfandyari S, Mokdad AH, Hotez PJ, Gasser RB. SARS-CoV-2 seroprevalence worldwide: a systematic review and meta-analysis. Clin Microbiol Infect. 2021 Mar;27(3):331-340.

31. Scheufele, D. A., and N. M. Krause. 2019. "Science Audiences, Misinformation, and Fake News." Proceedings of the National Academy of Sciences 116 (16): 7662–7669. doi:10.1073/pnas.1805871115.

32. Harmon-Jones E, Harmon-Jones C, Levy N. An Action-Based Model of Cognitive-Dissonance Processes. Current Directions in Psychological Science. 2015;24(3):184-189. doi:10.1177/0963721414566449

33. Bansal-Travers M, Hammond D, Smith P, Cummings KM. The impact of cigarette pack design, descriptors, and warning labels on risk perception in the U.S. Am J Prev Med. 2011;40(6):674-82. doi: 10.1016/j.amepre.2011.01.021.

34. Mays D, Niaura RS, Evans WD, et al. Cigarette packaging and health warnings: the impact of plain packaging and message framing on young smokers. Tobacco Control 2015;24:e87-e92.

35. Popova L, Owusu D, Jenson D, Neilands TB. Factual text and emotional pictures: overcoming a false dichotomy of cigarette warning labels [published online ahead of print, 2017 Apr 20]. Tob Control. 2017;tobaccocontrol-2016-053563. doi:10.1136/tobaccocontrol-2016-053563

36. Kornfield R, Smith KC, Szczypka G, Vera L, Emery S. Earned media and public engagement with CDC's "Tips from Former Smokers" campaign: an analysis of online news and blog coverage. J Med Internet Res. 2015;17(1):e12. Published 2015 Jan 20. doi:10.2196/jmir.3645

37. Bennett P, Noble S, Johnston S, et al COVID-19 confessions: a qualitative exploration of healthcare workers experiences of working with COVID-19. BMJ

For peer review only

Figure 1 - Outline of higher-order and lower-themes on COVID-19 communication strategies

Uploaded separately as PDF as per editorial office request.

For peer review only

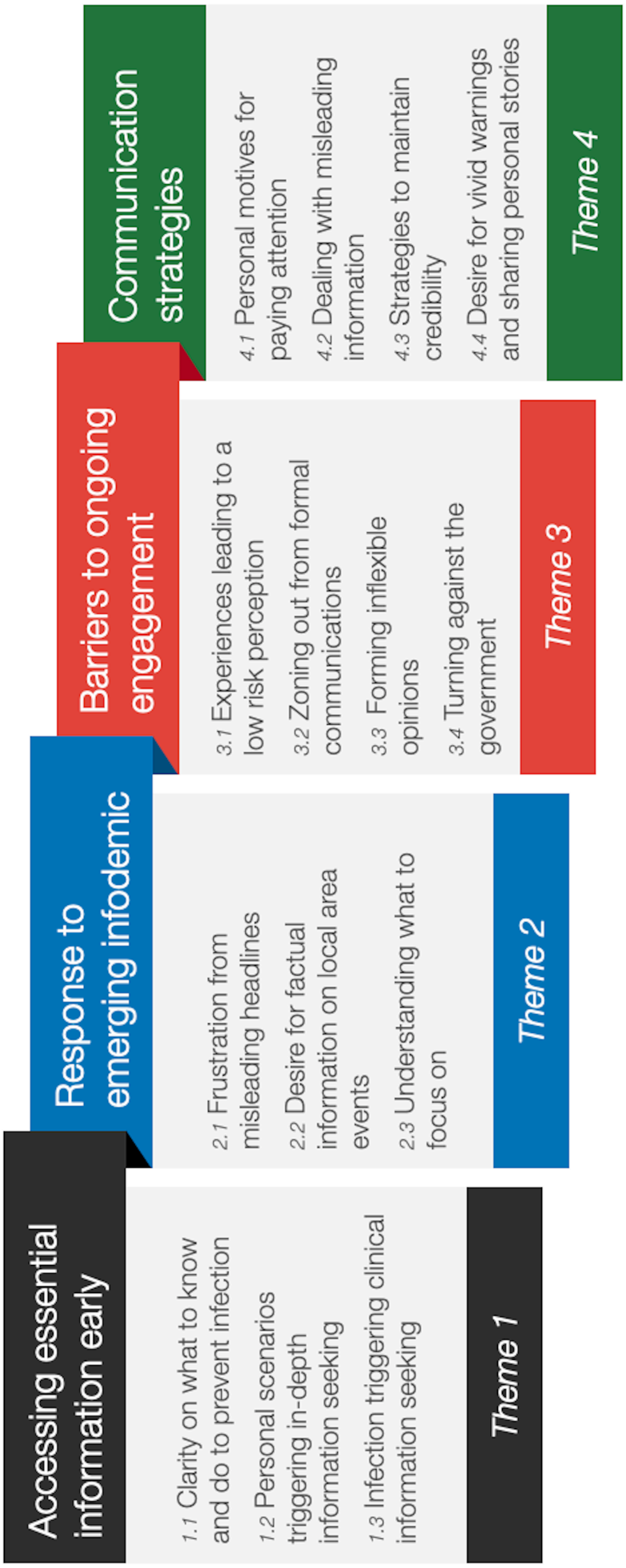
Theme 1
Accessing essential information early
1.1 Clarity on what to know and do to prevent infection
1.2 Personal scenarios triggering in-depth information seeking
1.3 Infection triggering clinical information seeking
Theme 2
Response to emerging infodemic
2.1 Frustration from misleading headlines
2.2 Desire for factual information on local area events
2.3 Understanding what to focus on
Theme 3
Barriers to ongoing engagement
3.1 Experiences leading to a low risk perception
3.2 Zoning out from formal communications
3.3 Forming inflexible opinions
3.4 Turning against the government
Theme 4
Communication strategies
4.1 Personal motives for paying attention
4.2 Dealing with misleading information
4.3 Strategies to maintain credibility
4.4 Desire for vivid warnings and sharing personal stories

Standards for Reporting Qualitative Research (SRQR)*

http://www.equator-network.org/reporting-guidelines/srqr/

Title and abstract

	Page/line no(s).
**Title** - Concise description of the nature and topic of the study Identifying the study as qualitative or indicating the approach (e.g., ethnography, grounded theory) or data collection methods (e.g., interview, focus group) is recommended	1
**Abstract** - Summary of key elements of the study using the abstract format of the intended publication; typically includes background, purpose, methods, results, and conclusions	3

Introduction

**Problem formulation** - Description and significance of the problem/phenomenon studied; review of relevant theory and empirical work; problem statement	5
**Purpose or research question** - Purpose of the study and specific objectives or questions	5

Methods

**Qualitative approach and research paradigm** - Qualitative approach (e.g., ethnography, grounded theory, case study, phenomenology, narrative research) and guiding theory if appropriate; identifying the research paradigm (e.g., postpositivist, constructivist/ interpretivist) is also recommended; rationale**	7
**Researcher characteristics and reflexivity** - Researchers' characteristics that may influence the research, including personal attributes, qualifications/experience, relationship with participants, assumptions, and/or presuppositions; potential or actual interaction between researchers' characteristics and the research questions, approach, methods, results, and/or transferability	8
**Context** - Setting/site and salient contextual factors; rationale**	6
**Sampling strategy** - How and why research participants, documents, or events were selected; criteria for deciding when no further sampling was necessary (e.g., sampling saturation); rationale**	6
**Ethical issues pertaining to human subjects** - Documentation of approval by an appropriate ethics review board and participant consent, or explanation for lack thereof; other confidentiality and data security issues	2
**Data collection methods** - Types of data collected; details of data collection procedures including (as appropriate) start and stop dates of data collection and analysis, iterative process, triangulation of sources/methods, and modification of procedures in response to evolving study findings; rationale**	5

For peer review only - http://bmjopen.bmj.com/site/about/guidelines.xhtml

For peer review only

**Data collection instruments and technologies** - Description of instruments (e.g., interview guides, questionnaires) and devices (e.g., audio recorders) used for data collection; if/how the instrument(s) changed over the course of the study	6
**Units of study** - Number and relevant characteristics of participants, documents, or events included in the study; level of participation (could be reported in results)	7
**Data processing** - Methods for processing data prior to and during analysis, including transcription, data entry, data management and security, verification of data integrity, data coding, and anonymization/de-identification of excerpts	6
**Data analysis** - Process by which inferences, themes, etc., were identified and developed, including the researchers involved in data analysis; usually references a specific paradigm or approach; rationale**	7
**Techniques to enhance trustworthiness** - Techniques to enhance trustworthiness and credibility of data analysis (e.g., member checking, audit trail, triangulation); rationale**	7

Results/findings

**Synthesis and interpretation** - Main findings (e.g., interpretations, inferences, and themes); might include development of a theory or model, or integration with prior research or theory	8
**Links to empirical data** - Evidence (e.g., quotes, field notes, text excerpts, photographs) to substantiate analytic findings	Table 1-4

Discussion

**Integration with prior work, implications, transferability, and contribution(s) to the field -** Short summary of main findings; explanation of how findings and conclusions connect to, support, elaborate on, or challenge conclusions of earlier scholarship; discussion of scope of application/generalizability; identification of unique contribution(s) to scholarship in a discipline or field	12
**Limitations** - Trustworthiness and limitations of findings	16

Other

**Conflicts of interest** - Potential sources of influence or perceived influence on study conduct and conclusions; how these were managed	2
**Funding** - Sources of funding and other support; role of funders in data collection, interpretation, and reporting	2

*The authors created the SRQR by searching the literature to identify guidelines, reporting standards, and critical appraisal criteria for qualitative research; reviewing the reference lists of retrieved sources; and contacting experts to gain feedback. The SRQR aims to improve the transparency of all aspects of qualitative research by providing clear standards for reporting qualitative research.

For peer review only

For peer review only - http://bmjopen.bmj.com/site/about/guidelines.xhtml

**The rationale should briefly discuss the justification for choosing that theory, approach, method, or technique rather than other options available, the assumptions and limitations implicit in those choices, and how those choices influence study conclusions and transferability. As appropriate, the rationale for several items might be discussed together.

Reference:

O'Brien BC, Harris IB, Beckman TJ, Reed DA, Cook DA. **Standards for reporting qualitative research: a synthesis of recommendations.** *Academic Medicine*, Vol. 89, No. 9 / Sept 2014 DOI: 10.1097/ACM.0000000000000388

For peer review only

For peer review only - http://bmjopen.bmj.com/site/about/guidelines.xhtml

BMJ Open

"Communication, that's the key": A qualitative investigation of how essential workers with COVID-19 responded to public health information

Journal:	*BMJ Open*
Manuscript ID	bmjopen-2022-061583.R1
Article Type:	Original research
Date Submitted by the Author:	06-Apr-2022
Complete List of Authors:	Roe, Mark; University College Dublin<br>Buggy, Conor; University College Dublin<br>Ingram, Carolyn; University College Dublin<br>Codd, Mary; University College Dublin<br>Buckley, Claire; Health Service Executive<br>Archibald, Mary; University College Dublin<br>Rachwal, Natalie; University College Dublin<br>Downey, Vicky; University College Dublin<br>Chen, Yanbing; University College Dublin<br>Sripaiboonkji, Penpatra; University College Dublin<br>Drummond, Anne; University College Dublin<br>Alvarez, Elizabeth; McMaster University, Health Research Methods, Evidence and Impact; McMaster University, Centre for Health Economics and Policy Analysis (CHEPA)<br>Perrotta, Carla; University College Dublin, School of Public Health, Physiotherapy and Sport Sciences
**Primary Subject Heading**:	Public health
Secondary Subject Heading:	Infectious diseases, Health policy, Communication
Keywords:	COVID-19, Public health < INFECTIOUS DISEASES, Health policy < HEALTH SERVICES ADMINISTRATION & MANAGEMENT

SCHOLARONE™
Manuscripts

BMJ

I, the Submitting Author has the right to grant and does grant on behalf of all authors of the Work (as defined in the below author licence), an exclusive licence and/or a non-exclusive licence for contributions from authors who are: i) UK Crown employees; ii) where BMJ has agreed a CC-BY licence shall apply, and/or iii) in accordance with the terms applicable for US Federal Government officers or employees acting as part of their official duties; on a worldwide, perpetual, irrevocable, royalty-free basis to BMJ Publishing Group Ltd ("BMJ") its licensees and where the relevant Journal is co-owned by BMJ to the co-owners of the Journal, to publish the Work in this journal and any other BMJ products and to exploit all rights, as set out in our licence.

The Submitting Author accepts and understands that any supply made under these terms is made by BMJ to the Submitting Author unless you are acting as an employee on behalf of your employer or a postgraduate student of an affiliated institution which is paying any applicable article publishing charge ("APC") for Open Access articles. Where the Submitting Author wishes to make the Work available on an Open Access basis (and intends to pay the relevant APC), the terms of reuse of such Open Access shall be governed by a Creative Commons licence – details of these licences and which Creative Commons licence will apply to this Work are set out in our licence referred to above.

Other than as permitted in any relevant BMJ Author's Self Archiving Policies, I confirm this Work has not been accepted for publication elsewhere, is not being considered for publication elsewhere and does not duplicate material already published. I confirm all authors consent to publication of this Work and authorise the granting of this licence.

For peer review only

"Communication, that's the key": A qualitative investigation of how essential workers with COVID-19 responded to public health information

Mark Roe[1], Conor Buggy[1], Carolyn Ingram[1], Mary Codd[1], Claire Buckley[2], Mary Archibald[1], Natalie Rachwal[1], Vicky Downey[1], Yanbing Chen[1], Penpatra Sripaiboonkji[1], Anne Drummond[1], Elizabeth Alvarez[3], Carla Perrotta[1]

[1] School of Public Health, Physiotherapy and Sports Science, University College Dublin, Ireland

[2] Health Service Executive, Dublin, Ireland

[3] Department of Health Research Methods, Evidence and Impact, McMaster University, Canada

Corresponding author
Mark Roe
School of Public Health, Physiotherapy, and Sports Science
Health Science Building
University College Dublin
Dublin
D04 V1W8
Ireland

Email: mark.roe@ucd.ie
Phone: +353 1 716 7777

Word count: 4,385

Tables: 4

Figures: 1

For peer review only

Patient and public involvement

Patients and/or the public were not involved in the design, or conduct, or reporting, or dissemination plans of this research.

Patient consent for publication

Not required.

Ethical approval

Ethical approval was granted by the UCD Human Research and Ethics Committee (ID: LS-E-21-85-Roe-Perrotta). Verbal informed consent was obtained from the study participants.

Provenance and peer review

Not commissioned; externally peer reviewed.

For peer review only

ABSTRACT

Objectives

To understand how essential workers with confirmed infections responded to information on COVID-19.

Design

Qualitative analysis of semi-structured interviews conducted in collaboration with the national contact tracing management programme in Ireland.

Setting

Semi-structured interviews conducted via telephone and Zoom Meetings.

Participants

18 people in Ireland with laboratory confirmed SARS-CoV-2 infections using real-time polymerase chain reaction testing of oropharyngeal and nasopharyngeal swabs. All individuals were identified as part of workplace outbreaks defined as ≥2 individuals with epidemiologically linked infections.

Results

A total of four high-order themes were identified: (1) Accessing essential information early, (2) responses to emerging "infodemic", (3) barriers to ongoing engagement, and (4) communication strategies. Thirteen lower-order, or subthemes, were identified and agreed upon by the researchers.

Conclusions

Our findings provide insights into how people infected with COVID-19 sought and processed related health information throughout the pandemic. We describe strategies used to navigate excessive and incomplete information and how perceptions of information providers evolves overtime. These results can inform future communication strategies on COVID-19.

For peer review only

STRENGTHS AND LIMITATIONS

- The qualitative design enabled the collection of data on the experiences and perceptions of people who had laboratory confirmed COVID-19 infections.
- Throughout the study the researchers used several best-practice strategies to complete thematic analysis of data from semi-structured interviews.
- Given the lack of prior qualitative research on this topic and cohort, we used inductive reasoning approach which enables us to avoid assumptions of prior research or frameworks.
- Participants were selected from a sub-group of the population known as 'essential workers' that could not avail of 'remote working' throughout the pandemic making their views are critical to informing public health policies that cater for cohorts at increased risk of infection.
- Our recruitment strategy had a low response rate perhaps indicating a lack of willingness in this cohort to engage with COVID-19 research or initiatives associated with official public health organisations.

For peer review only

BACKGROUND

Sharing public health information is a crucial step in creating awareness of threats and protective strategies.[1] The COVID-19 pandemic is unique in two ways. First, it is the first pandemic where digital technologies and social media platforms are used to share information to keep people safe.[2] Second, although little information was available on the novel SARS-CoV-2 pathogen in January 2020, an 'infodemic' had emerged within four months, prompting global responses such as the World Health Organization's Information Network for Epidemics (EPI-WIN).[3]

While acknowledging of wide-spread access to information on COVID-19, little is known about how people receive and process such content. This is especially true for groups at a higher risk of infection who are associated with super-spreading events (SSEs) which have a greater influence on the trajectory of epidemics.[4]

Prior research on COVID-19 information has explored the public's perceptions of communication strategies, and their ability to correctly answer questions on COVID-19 epidemiology.[5, 6] These offer little insight into how people who were most at risk of infection (e.g. high risk workers) make sense of information on COVID-19 or how they believe communication strategies could be improved in the future.

Using semi-structured interviews, this study aims to investigate how essential workers with confirmed infections responded to information on COVID-19. This research could inform workplace-related public health communications as the current pandemic continues and future pandemics emerge.

METHODS

This study represents a sub-analysis of a preregistered study to investigate the behaviours and contextual factors associated with COVID-19 outbreaks and SSEs (OSF: https://osf.io/aeg74).

Patient and public involvement

There was no patient or public involvement in setting the research agenda.

For peer review only

Data collection methods

Semi-structured interviews were conducted using a password-protected Zoom account via a secure login. Only the researcher and invited participant were admitted to the call. A transcript of the audio recording was generated and corrected following a playback of the original audio file, enabling the researchers to remove any identifiable information. Interviews lasted a combined total of 7.6 h (average 25.2 ± 12.5 min) generating 45,987 words. Each participants was assigned a unique identification number (PID). After this process, recordings were deleted from all sources. Four researchers completed the interviews following a series of pilot interviews with fellow team members (NR = 8; MR = 6; MA = 2; CI = 2). All interviews were conducted from 22nd July 2021 to 7th Aug 2021.

For context, as of 22nd July 2021, Ireland reported 289,139 laboratory confirmed infections since the first case on 29th February 2020, equating to a population prevalence of 5.8%.[7] The median age of national cases was 22. Healthcare workers accounted for 10.3%. A total of 15,134 hospital admissions and 5,026 were reported. Approximately, 63.8% and 53.0% of the population had been partially or fully vaccinated against COVID-19. In the 14-days prior to data collection, Ireland confirmed 13,065 infections (prevalence = 0.3%) with 29.5% of cases associated with community transmission.[8] The median age was 23. A similar infection rate was reported until 13th Oct 2021. Ireland's 7-day test positivity rate was 5.9-7.9% throughout the study period.[7]

We developed an interview guide to explore three areas: behaviours and contextual factors leading to COVID-19 outbreaks, responses to minimise transmission, and lessons for future responses. These areas were identified following a review of the literature, consultation with national public health authorities, and initial findings from a larger COVID-19 research project by the authors (Science Foundation Ireland: 20/COV/8539). Within this we asked specific questions on information and public health messaging and the role of the media in minimising transmission and sustaining safe behaviours. Prompt questions were asked by the researchers to illicit more detailed information on these topics.

For peer review only

Study participants

We recruited 18 people in the Republic of Ireland who had laboratory-confirmed SARS-CoV-2 infections using real-time polymerase chain reaction testing of oropharyngeal and nasopharyngeal swabs. All individuals were identified as part of suspected workplace outbreaks where ≥2 individuals had epidemiologically linked infections. We targeted this group given their first-hand experience of (1) COVID-19 outbreaks, and (2) responses to minimise transmission. Therefore, inclusion criteria included age ≥18 y and a laboratory confirmed infection likely linked to a workplace outbreak.

Potential participants were selected from a sub-group of the population known as ‘essential workers’ that could not avail of ‘remote working’ throughout the pandemic. Potential participants (n=167) were identified by the partner Health Service Executive (HSE) Contact Tracing Centre (CTC) as fulfilling the inclusion criteria, and during routine surveillance activities, were invited to receive a call from the research team. Overall, 104 individuals were contactable by the HSE CTC (62.3%). From this cohort, 64 (61.5%) refused to be contacted by the research team while 40 (38.5%) gave consent. We were able to contact and complete interviews with 18 people from this sample, representing 10.8% of potential participants originally identified by the HSE CTC.

Seven females (38.9%) and 11 males (61.1%) were interviewed. The median age was 35 y (min = 18; max = 57). Sectors represented included food, construction, laundry, retail, energy, healthcare, professional services, public services, and emergency services.

Data analysis

We used a thematic analysis approach to support a structured exploration of patterns in perceived behaviours and contextual factors associated with COVID-19 outbreaks experienced by the participants.

The coders (MR x 10, VD x 8) familiarised themselves with the transcripts prior to initiating coding. Thematic analysis was conducted in accordance with the six phases of trustworthy thematic analysis as outlined by Nowell et al. (2017) (i.e. familiarisation

For peer review only

with the data; generating initial codes; searching for themes; reviewing themes; defining and naming themes; producing the report).[9] Qualitative analysis software (NVivo V12) was used to transform raw data into multiple codes using open and axial coding processes. A final of 35 codes were computed.

We considered saturation was met when two conditions were realised: (1) agreement between researchers that no further transcript codes could be generated, and (2) no further information, deemed relevant to the stated research questions, could be obtained from the transcripts.[10] Discrepancies were resolved through discussion until a consensus was reached. Reflective discussions were conducted (MR, VD, CI) throughout the study to review the rationale for selecting codes, themes, and quotes.[11] Credibility strategies used throughout analysis included (1) critical friend approach by encouraging reflexivity by asking provocative questions to critique our research in a supportive manner that clarifies ideas and interpretation, and (2) cross-checking of themes identified in prior focus group studies.[12] The team has a diverse range of background (e.g. medicine, public health, epidemiology, risk management, occupational health, psychology) that facilitated a robust review of identified themes. Results are reported in line with SRQR reporting guidelines (Supplementary file 1).[13]

RESULTS

A total of four high-order themes were identified: (1) Accessing essential information early, (2) responses to emerging infodemic, (3) barriers to ongoing engagement, and (4) communication strategies. Thirteen lower-order, or subthemes, were identified and agreed upon by the researchers (Figure 1).

Theme 1: Accessing essential information early

The first theme highlights that participant reported access to information on the essential elements of COVID-19 before a pandemic was first declared in March 2020. The identified sub-themes reveal the role of clear communications and personal thresholds for seeking information to reduce transmission of the virus in work settings.

For peer review only

Sub-theme 1.1: Clarity on what to know and do to prevent infection

Participants expressed the view that since COVID-19 was first confirmed in Ireland on 29th February 2020, public health information was almost unavoidable given the extent of coverage on news programmes and social media. Most participants accessed information via national television news shows which included live broadcasts of public health press briefings and newspapers as well as websites of the HSE and World Health Organization. Additionally, every participant reported having access to information on COVID-19 in their workplace. Workplace information provided clarity on what staff could expect if workers developed symptoms of COVID-19. Interestingly, it appears that prior to their infection, participants largely tended to only seek generic information on COVID-19 that was deemed necessary for functional tasks (table 1).

Sub-theme 1.2: Personal scenarios triggering in-depth information seeking

Participants referred to triggers that initiated searches to answer specific queries related to COVID-19 that arose from unique personal events (table 1). This was largely indicative of personal concerns related to changes in their own health status, or that of an acquaintance or family member, leading to a reappraisal of the threat posed by COVID-19. More in-depth information was sought from specific Google searches, YouTube videos, and formal research papers. It appears these intentional information seeking activities served the specific purpose of helping the participants make sense of their personal scenarios.

Sub-theme 1.3: Infection triggering clinical information seeking

We also noticed that even following confirmation of a positive test, participants only sought information when COVID-19 began to impact their health (table 1). A clear distinction was noted between this and sub-theme 1.2, as information seeking occurred after a specific event (i.e. following confirmed infection) and refers to the specific domain of case management (i.e. what are the potential clinical outcomes, and how what actions can support better outcomes). Participants expressed that these searches for specific clinical information allowed them to prepare for what might be expected to occur throughout their period of self-isolation.

For peer review only

Table 1 – Illustrative quotes supporting theme one 'accessing essential information early'

Sub-Theme	Supporting Quotes	Participant ID
1.1 Clarity on what to know and do to prevent infection	You were kind of just told what the symptoms were, and you were educated on what symptoms to look out for and if you have any of these symptoms to report them and to isolate at home.	PID 12: Nurse
	Everyone was already pretty much informed of what would happen if there was a scenario where someone gets sick.	PID 17: Meat plant worker
	The procedures were rolled out nationally. There was no issue with dissemination of information. The quality of information coming at a national level, it was good.	PID 20: Emergency services worker
	There's a lot of access [to information] in every part of the company. [It's been] very very good through the COVID period so I give out credit for that.	PID 7: Food factory worker
	I got the information I needed, I never looked for any more information that I didn't need.	PID 12: Nurse
1.2 Personal scenarios triggering in-depth information seeking	It's [not] until either they get COVID or they see someone that they know get COVID badly, once that happens then they'll realize what COVID is, and that's the sad reality is that there's a lot of people who won't take this thing seriously until they see somebody that they know with COVID.	PID 1: Meat plant worker
	When I got the headache I obviously was looking up to see was this part of the symptoms. Is it actually COVID or is it just this cough I had before Christmas? So I definitely was seeking out symptom information, and I read, I don't know it was like a dissertation or a there was a paper written on this 72 hour headache that wouldn't react to any medication or anything so that kind of rang true.	PID 16: Office worker in professional services
1.3 Infection triggering clinical information seeking	I won't really be looking for information unless I like start showing symptoms and I start to feel very unwell then I'll start looking at it going like, right, what is it, how can I help myself with this, what can I do.	PID 12: Nurse
	[At the time] I was just kind of reading through different information about symptoms and how long they last and different things that can happen.	PID 16: Office worker in professional services

For peer review only

Theme 2: Response to emerging infodemic

The second theme emerged in response to participants frequently discussing the rapid growth of information on COVID-19 despite it often being incomplete, and at times, misleading or of little use to inform their personal actions. Here we noticed a desire for information based on how the COVID-19 epidemic was progressing in local areas.

Sub-theme 2.1: Frustration with misleading headlines

We repeatedly found references to perceived over-reporting on COVID-19 by the media (table 2). Whilst participants mentioned that although the sustained media coverage was at times fear-provoking, it also played a useful role in keeping the public informed of the latest COVID-19 trajectory. Participants reported being disheartened by the manner of reporting, as opposed to the constant publication of media content related to COVID-19. Similarly, participants also alluded to their distaste for some marketing tactics, deployed by some media outlets to increase online traffic, which had the potential to misinform the public.

Sub-theme 2.2: Desire for factual information on local area events

Our analysis identified that participants valued information related to their local geographical area, going beyond the reporting of mere confirmed case numbers to reveal details of infection sources and clinical outcomes (table 2). This was seen as beneficial to raising awareness of the threat in the area in attempts to bolster adherence to infection control measures.

Sub-theme 2.3: Understanding what to focus on

Despite repeated references to excessive amounts of information on COVID-19 in the media and online, participants appear to have coped by considering the relevance of the content for understanding the nature of the virus, and how it could inform their future actions (table 2).

For peer review only

Table 2 – Illustrative quotes supporting theme two 'response to emerging infodemic'

Sub-Theme	Supporting Quotes	Participant ID
2.1 Frustration with misleading headlines	I feel like the cases are kind of annoying hearing about [them] every day, but I feel like it is good to know… I think they just report on it too often kind of scaring people like as in they kind of use headlines to scare people, when you read the actual information it's very misleading.	PID 14: Factory worker
	Give proper factual information instead of kind of click bait headlines on everything.	PID 14: Factory worker
2.2 Desire for factual information on local area events	There should be maybe more information on it like how many people are like in hospital in each area and like where the cases are explicitly coming from. [I] think it'd be more beneficial than maybe just hearing the cases every day, you kind of stop listening and stop caring, there's like 1,000 cases when like 4 million people like it doesn't, but if you kind of read it and know people are near you it might make people pay more attention and stick to the rules and keep safe	PID 14: Factory worker
	I think the government needs to be more transparent on things and provide more accurate information because at the time when this whole pandemic was rampant I feel like the government was just overloading people with so much information that you didn't even know which one is right which one is wrong. You could be saying 'oh that's what I heard is' and then another person could be saying 'oh no, this is what I heard is' and like you know it's all a bunch of everything. All the information is all jumbled up and the truth is somewhere in the middle.	PID 17: Meat plant worker
2.3 Understanding what to focus on	It's just knowing what this virus is and knowing how it's going to affect your body and being able to prepare yourself for when it's going to affect your body	PID 1: Meat plant worker
	I was just reading more about it and what it actually does and how or what I'm supposed to take or do to make my immune system stronger, so those were the things I was checking	PID 3: Laundry worker
	I guess the main thing is have information readily available for everybody just to keep on track and keep updated on what the most recent guidelines are.	PID 12: Nurse

For peer review only

Theme 3: Barriers to ongoing engagement

Theme three may be best conceptualised as the consequences of participants having to seek and process information on COVID-19 in a largely ad-hoc personally motivated manner (theme 1) whilst dealing with the factors of incomplete and context-free information (theme 2). We heard how this led some essential workers to reduce their perception in the threat of COVID-19 whilst simultaneously changing the nature of their engagement with official sources of public health information. Within this process, social media sites became platforms playing an ever-increasing role in re-forming opinions of COVID-19 as well as the government and officials responding to it. This was rooted in a perceived mismatch between information reported on COVID-19 and insights gained from first-hand experience of being infected, resulting in some participants stating that they will be less likely to engage with communications in the future.

Sub-theme 3.1: Experiences leading to a low-risk perception

We noted that several participants, having reflected on how the congruency between their personal experiences throughout the pandemic and those reported by the media, expressed a current belief that COVID-19 was not as dangerous as reported in the media (table 3). These participants believed that they correctly understood the content of prior media reports, yet witnessed, or heard about, contradictory COVID-19 outcomes prior to adopting their alternative beliefs. A source of evidence for personal experiences leading to a lower risk perception appears to be perceived wrong information that was reported yet never acknowledged or corrected. We noted that such participants were eager to emphasise that they were not denying the existence of COVID-19.

Of 18 participants, we encountered only one that alluded to conspiracy theories. Thus, we draw a clear distinction between the emergence of alternative hypothesises, at odds with the narratives reported by official authorities or media outlets, and conspiracy theories citing malicious motives by the official organisations.

For peer review only

Sub-theme 3.2: Zoning out from formal communications

Participants referred to a reduced ability to continually engage with media and public health messages over time, resulting in unintended outcomes such as when some people become "accustomed to hearing about it and stop caring". Formal communications strategies (i.e. content released by national health authorities such as press briefings) on COVID-19 in Ireland were first implemented in February 2020. We noted the belief that the public developed a reduced willingness, or capacity, to engage in this form of messaging as the pandemic progressed (table 3). Instead, participants mentioned the role of 'informal advertisements', associated with the placing of public health messages during the start and end of popular programs and delivered by the host.

Sub-theme 3.3: Forming inflexible opinions and turning against the government

Participants recognised the role of social networks, particularly those online, in shaping peoples' interpretation of information related to COVID-19. Specifically, we noted the perception that online social networks were effective at facilitating a process whereby users arrived at an interpretation of COVID-19 information that was more likely to be anti-establishment (table 3).

Participants spoke of the consequences of prior measures taken to reduce transmission of COVID-19. In particular, national lockdowns, known locally as 'Level Five restrictions' were identified as being a source of anger amongst the public. We found that participants were unlikely to dissociate government officials from senior members of Ireland's National Public Health Emergency Team. Additionally, there was a sentiment that the public's negative reaction to previous COVID-19 responses would lead some people to adopt a negative attitude of future messages regardless of the content (table 3).

For peer review only

Table 3 – Illustrative quotes supporting theme three ‘barriers to ongoing engagement’

Sub-Theme	Supporting Quotes	Participant ID
3.1 Experiences leading to a low risk perception	I believe soon it’s coming, another lockdown. Soon, very soon I believe, and I think that you know that already. There are so many cases that’s why. Because I said like I don’t know how [they're] can be so many cases. But we never... heard, like we saw on the news there are few cases like 900 or something like that a day... but we didn’t hear like somebody had for real COVID.	PID 22: Meat plant worker
	It’s not like we don’t believe about COVID, alright it’s a virus I understand, but it’s not as worse [as] they actually say… How can you believe everything they say on the news and everything when people every day see different?	PID 21: Construction worker
3.2 Zoning out from formal communications	So the only information I really looked for was [when symptomatic], I was talking to my boss at the time so he was the one who was keeping me informed with what I should be doing at all, so uh he told me to stay off. I think it was 2 days and then if I still had symptoms or if symptoms [hadn't] got better I was to get a test at the GP.	PID 17: Meat plant worker
	I feel like some people when they kind of read the news like what will make them be more careful, but sometimes it’s the opposite. Like if people are hearing it every day they just kind of just become accustomed to hearing about it and stop caring.	PID 13: Nurse
	You always hear [TV host] when he’s on [Ireland’s most popular entertainment show] wrapping it up saying “everyone make sure to wear your masks and wash your hands”, so if it was kind of embedded into more of an informal advertisement. I think it could be responded to better or even like on the radio channels just that it's nearly embedded subconsciously into people. But I think the formality on how they’re doing it now, it’s just lost the interest of people. People are just zoned out they don’t care anymore about it.	PID 13: Nurse
3.3 Forming inflexible opinions and turning against the government	People are very headstrong. They'll keep this thought that they have of whatever their opinion on COVID is because, like, they'll have one or two people agree with them on Facebook or something, and they’ll think alright well what I’m saying is correct.	PID 1: Meat plant worker
	Some people like they read too much online on Facebook, on whatever like Instagram or whatever. And then [get] sucked into it and then they end up believing it, and then it just keeps going on and on until they're sitting there telling people that COVID isn’t real or that the vaccine is trying to control them. But, like, it's just that some people think that anything they're told shouldn't be taken at face value. Some people find it hard that when the government says something, that’s the truth, some people do find that very hard.	PID 1: Meat plant worker
	The main thing is, especially with the measures that we had in place when we were in Level Five, I think those were very, they were very harsh. I understand. Yet it did work. But it didn't really. Like, there were a lot of people that turned against the government.	PID 1: Meat plant worker
	I think to be honest with ya a lot of people have a negative response to the government and most of the directors I’ve heard from the government. I know a lot of people don’t like [Ireland’s Chief Medical Officer]. I don’t mind him, but a lot of people don’t like him, and if they say something they’re [of] the attitude that they're not just going to take it on board, they’re just going to be disrespectful to him and it’s just going to ignored from the get go.	PID 13: Nurse

For peer review only

Theme 4: Communication strategies

The final theme we identified related to how participants believed that future communication strategies on COVID-19 and future public health emergencies could be improved.

Sub-theme 4.1: Personal motives for paying attention

Despite referring to excessive information and media coverage, all participants appeared to value ongoing communication. Similar to our findings in sub-theme 1.2 (i.e. personal scenarios triggering in-depth information seeking), participants expressed a need for future communications to be anchored to a personal value proposition, such as protecting loved ones (table 4).

Sub-theme 4.2: Dealing with misleading information

All participants used social media yet were unclear about specific measures that could be taken to reduce the misinformation on digital platforms (table 4).

Sub-theme 4.3: Strategies to maintain credibility

Our analysis indicates that to avoid misinformation, participants adopted a strategy of seeking information from several recognised health authorities, rather than a sole source, before cross-checking the content with media reports (table 4).

Sub-theme 4.4: Desire for vivid warnings and sharing personal stories

When considering lessons for future strategies to improve adherence to public health measures, participants again referred to personal scenarios. Specifically, strategies used to respond to promote awareness of other public health threats, such as smoking, were mentioned by participants (table 4).

For peer review only

Table 4 – Illustrative quotes supporting theme four 'communication strategies'

Sub-Theme	Supporting Quotes	Participant ID
4.1 Personal motives for paying attention	Communication that's the key to explain to them, to explain things to them because if you love someone, your parent, if your parent is vulnerable, you need to protect them, we protect each other… if I protect myself, I protect also my family… If we pay attention [to] the information, we can protect each other.	PID 4: Laundry worker
	The information being disseminated is continued and is of good quality but that depends on the individual person, whether they decide to look at that information or read it. There was no onus on anybody to read it and if they didn't it wouldn't make a whole lot of difference to be honest with ya.	PID 20: Emergency services worker
	I think some people, they should get more information when we know [that COVID-19] is really happening in the world. So me and my daughter and my family we got [the] most information we could. But all the time we leave we put mask on, alcohol gel we have in handbag, all the time using sanitizer to clean the doors and stairs and everything. But when I start work, I see some people they doesn't care so much, you know.	PID 7: Food processing plant general manager
	I mean one key piece of information that I'd say is make sure people know that in the case of testing positive they won't go without pay. Because like I can see that causing a lot of unrest with people because you know it's like I said, a lot of people have families to feed you know and people rely on them for the money to get by. If people knew that and it's like 'oh I'll be taken care of if this happens' you know, they'll be a lot more willing to cooperate with the normal guidelines.	PID 17: Meat plant worker
4.2 Dealing with misleading information	Maybe if there was something in place to kind of stop all the false news and kind of stuff that's spread online.	PID 14: Factory worker
	[When tested positive] the HSE [national health service] send me email for ten days about what I do [and to ask] how I feel.	PID 9: Meatplant worker
4.3 Strategies to maintain credibility	All the guys are doing everything that [they] can do because there's so much more information about this... Because everything we know we get it from HSE [Health Service Executive], we get it from news, we get it from TV. Like everything, we get it from information from the health organisations giving to us.	PID 7: Food processing plant general operations
	It's just about reinforcing the information, the guidelines. It's a bit like any other training that we do in healthcare when it comes to basic life support or infection control or anything like that. We do annual updates of that every year and that is the simple purpose to keep the practice up, to keep your skills up and to keep it on the forefront of your mind so that you don't kinda forget and let standards slip. I think that's really the best way really to go forward, is to have support if you have any questions and have that support readily available but as well as that just having the information there and having regular updates and what's best to do.	PID 12: Nurse, female
	[When infected] I had access from the [contact tracing caller] to all the information. I feel like they had all the information.	PID 8: Meatplant worker

For peer review only

4.4 Desire for vivid warnings and sharing personal stories	They should have a picture of what COVID does to your body and they'll help people realize because, like, on the smoking packets, it does help people realise and they say on them like the dangers of smoking. It shows the harsh reality of it, and that's what people need to realise is the harsh reality.	PID 1: Meat plant worker
	From a public point of view, all I can say is again information. Just that information is put out there readily for everyone. That it's clear, it's concise, it's honest, and it's blunt with regards to the impact that it will have and what's expected.	PID 12: Nurse
	Like I got infected. I could tell them, 'this is my story and yes it [is] real. You should believe this'.	PID 3: Laundry worker
	Thank you for calling and thank you for letting me explain my experience because it's very hard when you are alone. Like I'm single mother, just me and my daughter, so if something happen with me I was scared with who was going to look after my baby and all this things. But at same time [being infected] make me more strong, make me look ahead and say listen 'this is going to pass, this is not going to affect you forever, this is just a moment, uh, so keep calm'.	PID 7: Food processing plant general operations

For peer review only

DISCUSSION

Our study investigated how essential workers with confirmed infections responded to public health information on COVID-19. Analysis of semi-structured interviews with 18 participants identified four themes: (1) accessing essential information early, (2) response to emerging infodemic, (3) barriers to ongoing engagement, and (4) communication strategies. No hypothesis was tested. Instead, we used inductive reasoning to explore patterns in COVID-19 information usage, identifying several findings that can inform public health information strategies.

The precursor for seeking information is a perceived need to understand (i.e. describe what is) and respond (i.e. prescribe what should be done) to an unfolding event (i.e. COVID-19).[14] These needs evolve overtime, are directed by personal experiences, and determine the perceived importance of seeking the best possible information or making correct decisions.[15]

Given the extent of coverage in the media, information on COVID-19 was practically unavoidable in 2020. Early information on COVID-19 appears to have had the effect of focusing attention on symptoms and how suspected cases should be managed. These early ‘fundamental truths’, even amid incomplete information, were simple to process and capable of informing public behaviours.[16] This is important as most of the public in the region of study has had no prior experience of pandemic pathogens.

We identified that most participants obtained information from official organisations such as national and international health organisations, indicating that the people interviewed in the current study saw these sources as credible, or at least, worth seeking guidance from. In some instances, failure to share the best available information led people to seek info from misleading sources as described in subthemes 2.2 *desire for factual information on local area events,* and 3.2 *zoning out from formal communications*. Disengagement is happening and can be problematic. Yet as it happens, we see examples of lived experiences contributing to healthy behaviours. Thus, a solution might be to facilitate exchanges between producers of information and the general population to (1) ensure that needs and population expertise are being heard and (2) encourage engagement by building trust through dialogue and providing ongoing explanation.

For peer review only

Prior studies provide evidence that the perceived risk of infection depends on the proportion of neighbours that are ill, meaning that amid a lack of official communication on local events, people turn to personal networks to gain information on their neighbourhood.[17] In the era of digital networks, this inevitably funnels people towards social media platform, increasing exposure to misinformation and polarising opinions.[18]

We note calls for social media companies to regulate information deemed inaccurate, false, or malicious.[19] However, that is unlikely to satisfy information needs as people will most likely default to word of mouth.[20] For instance, Lupton and Lewis (2021) noted that the public use informal communications with friends and families, local and internationally, to keep up to date with emerging information.[21]

Whilst the role of clickbait headlines and social media is undoubtedly playing a role in spreading misinformation, so too is the inability of public health authorities to collect and disseminate information to people on live outbreaks in their communities. Surveillance capacities are a key component of the International Health Regulations for preparing and responding to infectious disease threats.[22] Tools such as community outbreak alerts might be particularly useful, especially as attention to specific diseases may decline after surges of infection when relatively low national figures are communicated.[17]

Although tools such as community outbreak alerts are common in many jurisdictions, Irish health authorities have not used them citing concerns related to data privacy, however, guidance from the national Data Protection Commissioner on 6th Mar 2020 states that “Article 9(2)(i) of [General Data Protection Regulations] GDPR and Section 53 of the Data Protection Act 2018 will permit the processing of personal data, including health data, once suitable safeguards are implemented. Such safeguards may include limitation on access to the data, strict time limits for erasure, and other measures such as adequate staff training to protect the data protection rights of individuals”.[23]

It is foreseeable that people receiving information at odds with their personal experiences creates doubts in credibility of sender. Our results imply that overtime, participants adopted a cautious attitude or scepticism towards information shared by

For peer review only

official and government organisations, indicating a degree of frustration and mistrust.[24]

Gaps in information lead to more reliance on experiential knowledge, a recognised useful resource when responding to uncertainty (e.g. learning from community leaders or professional mentors) especially if derived in settings that are difficult for outsiders to grasp.[25, 26] This is likely attributable to concerns about COVID-19, and the response to it, 'bubbling up' slowly overtime as the pandemic progresses.[27] Factors contributing to these changes in personal risk perceptions included delays in collecting and reporting information, the presence of random noise in that information, and the shared belief in social networks that the posed risk is reducing over time.[28] Hence, we observed an interplay between people making an effort to stay informed whilst simultaneously withdrawing disengaging from previously trusted sources, leading to emergence of more settled, and potentially alternative, beliefs running counter to official interpretations.[24] We believe this is also evident in our ability to only reach 10.8% of our identified sample.

Adopting beliefs by processing imperfect information through social interactions indicates the use of well-documented heuristic principles, mainly the representativeness heuristic (i.e. comparing risk situations with those that seem similar), the availability heuristic (i.e. assessing risk with information most easily accessed), and the anchoring heuristic (i.e. risk estimate based on previous event).[15, 28] Whilst this may seem undesirable to evidence-based domains, heuristics and risk management principles continue to form the basis of many effective COVID-19 measures that are defendable even in the absence of scientific evidence.[29, 30]

Additionally, the true risk of COVID-19 events has been difficult to accurately assess and provide information on. For example, as evidenced by large differences between reported case numbers and seroprevalence studies.[31] Overtime, as dissatisfaction manifests and trust in the information sender is eroded, it is unlikely that responses such fact-checking will deliver an immediate change in perception or behaviour.[32] In line with the action-based model of cognitive-dissonance processes, the sensation leading to a "zoning out", signalling a distancing from pre-trusted sources, as

For peer review only

inconsistencies in information and cognition must be resolved through alternatives means for the person to take logical course of action.[33]

Hence, it is oversimplistic to label ‘zoning out’ as a symptom of a catch-all term like pandemic fatigue (i.e. a perceived exhaustion of cognitive and emotional capacities to sustain the effort required to positively respond to outbreaks), given that it is influenced by a complex two-way sender-receiver process of detecting, disseminating, and deciphering key data, including outcomes of policies adopted by public health officials and government. Instead, it is perhaps more accurate to consider ‘zoning out’ as a stage in response to incomplete information from formal releases from state agencies that leads people to move away from previously reliable sources to seek additional information.

Participants referenced previous public health campaigns that involved graphic health warnings on cigarette packaging. Research has shown that the public have a preference for larger, pictorial, and loss-framed warning labels given that these are more likely to attract attention and further thought or information seeking on health risks.[34] Such communication strategies have been shown to be particularly effective at generating motivation to change behaviour in cohorts aged 13-30 y; a group more likely to online social media.[35, 36]

Ultimately, this emphasises the importance of message content (i.e. what to say) and message executions (i.e. how to say it) when communicating information on COVID-19.[37]. The need for reiterative approaches to develop public health communications has been highlighted and largely involves monitoring perceptions of message receivers of classic message inputs such as who, says what, through which channel, to whom, and with what effect.[37, 38] However, regardless of the message executions or framing, our study found that interviewed participants were sceptical about the underlying assumptions of message content (e.g. accuracy of information being communicated, or the relevance to their local community).

We also note similarities between our findings on communication strategies and those from a thematic analysis of social media posts linked to a video-based quit smoking campaign that exposed the public to comments deemed both oppositional (e.g. role of government, bigger perceived threats, scientific scepticism) and supportive (e.g.

personal stories, support for change, reactions to ad content).[39] Critics of graphic health warnings often believe that these strategies are emotional and not factual, however, evidence suggests that there is little difference in emotional response to information presented in graphic or factual text form, suggesting a false dichotomy.[36]

Whilst sustained communication can overwhelm, it is also a valued strategy to alleviate anxieties and uncertainty. For messages to provide clarity they must recognise both the change in information and misinformation.[21] This is because the public recognise that COVID-19 misinformation is common and expect scientists and governments to provide accurate information.[21]

Given the ever-evolving nature of infectious pathogens, the rapid detection and dissemination of health threats are heavily reliant on IT infrastructure for two-way communication between health providers and the public.[22, 24]

We also note references to sharing stories by participants. Like Bennett et al., we believe that future communication strategies could learn from personal experiences of people infected with COVID-19 if given “an outlet [to hear their] uncensored stories”.[40]

STRENGTHS AND LIMITATIONS

Our novel study design involved a partnership with the national contact tracing management programme. The qualitative design enabled the collection of data on the experiences and perceptions of people who had laboratory confirmed COVID-19 infections. Throughout the study the researchers used several best-practice strategies to complete thematic analysis of data from semi-structured interviews. Additionally, given the lack of prior qualitative research on this topic and cohort, we used inductive reasoning approach which enables us to avoid assumptions of prior research or frameworks.

A strength of our approach is the focus on essential workers given their repeated exposure to higher-risk settings throughout the pandemic, particularly when stay at home orders were imposed for the rest of society. This allows us to capture unique experiences of essential workers who were at the coalface of dealing with the threat of COVID-19 before widespread vaccination in society. It is likely that these shaped

For peer review only

participants perceptions of national responses to COVID-19 as outlined in theme three.

A potential limitation of this study is that our recruitment strategy had a low response rate perhaps indicating a low willingness in this cohort to engage with COVID-19 research or initiatives associated with official public health organisations. This may bias our sample and could be addressed through future contact tracing strategies. Additionally, our sample size (n=18) means our results cannot be generalised to the population or workforce as a whole. However, as participants represented essential workers, awareness of their views are important to inform public health policies that cater for cohorts at increased risk of infection.

For peer review only

Contributorship Statement

MR, CI, MA, NR, CP, MC, CB, CB, YC, PS, AD, and EA conceptualised and designed the study, and reviewed and revised the manuscript. MR, CI, MA, NR, CP, MC, CB, and CB designed the data collection instruments and recruited participants. MR, CI, MA, and NR conducted qualitative interviews. MR, VD, and CI analysed qualitative data. MR and CI drafted the initial manuscript. All authors approved the final manuscript as submitted and agree to be accountable for all aspects of the work.

Competing interests

None declared.

Data availability statement

Data are available on reasonable request. Raw transcripts of the data analysed in the study are available from the lead author.

Funding

This work was supported by Science Foundation Ireland (SFI) through the COVID-19 Rapid Response Research and Innovation Programme, grant number 20/COV/8539.

For peer review only

References

1. Mheidly N, Fares J. Leveraging media and health communication strategies to overcome the COVID-19 infodemic. J Public Health Policy. 2020;41(4):410-420. doi: 10.1057/s41271-020-00247-w.

2. Fagherazzi G, Goetzinger C, Rashid MA, Aguayo GA, Huiart L. Digital Health Strategies to Fight COVID-19 Worldwide: Challenges, Recommendations, and a Call for Papers. J Med Internet Res. 2020;22(6):e19284. doi: 10.2196/19284.

3. World Health Organization: About EPI-WIN. Accessed at: https://www.who.int/teams/risk-communication/about-epi-win

4. Broomell S, Kane P. Perceiving a pandemic: Global–local incompatibility and COVID-19 superspreading events. Decision, 2021;8(4), 227–236. https://doi.org/10.1037/dec0000155

5. Malecki KMC, Keating JA, Safdar N. Crisis Communication and Public Perception of COVID-19 Risk in the Era of Social Media. Clin Infect Dis. 2021;72(4):697-702. doi:10.1093/cid/ciaa758

6. Jaber RM, Mafrachi B, Al-Ani A, Shkara M. Awareness and perception of COVID-19 among the general population: A Middle Eastern survey. PLoS One. 2021;16(4):e0250461. doi:10.1371/journal.pone.0250461

7. Government of Ireland and Ordnance Survey Ireland: Ireland's COVID-19 Data Hub. Accessed at: https://covid-19.geohive.ie/search

8. Health Surveillance Protection Centre: Epidemiology of COVID-19 in Ireland: HPSC interim 14 day report on cases uploaded to the COVID Care Tracker. Accessed at: https://www.hpsc.ie/a-z/respiratory/coronavirus/novelcoronavirus/surveillance/covid-1914-dayepidemiologyreports/2021/july2021/COVID-19_Interim%2014%20day%20report_INTERIM_20210722_WEBSITE.pdf

9. Nowell LS, Norris JM, White DE, Moules NJ. (2017). Thematic Analysis: Striving to Meet the Trustworthiness Criteria. International Journal of Qualitative Methods. https://doi.org/10.1177/1609406917733847

10. Fusch PI, Ness LR. Are We There Yet? Data Saturation in Qualitative Research. The Qualitative Report 2015;20(9):1408-1416. https://doi.org/10.46743/2160-3715/2015.2281

11. Ortlipp M. Keeping and using reflective journals in the qualitative research process. Qual Rep 2008;13:695–705.

12. O'Brien BC, Harris IB, Beckman TJ, Reed DA, Cook DA. Standards for reporting qualitative research: a synthesis of recommendations. Acad Med. 2014;89(9):1245-1251.

13. Smith B, McGannon K. Developing rigor in qualitative research: problems and opportunities within sport and exercise psychology. International Review of Sport and Exercise Psychology. 2018;11(1):101-121. doi: 10.1080/1750984X.2017.1317357

14. Kim HK, Ahn J, Atkinson L, Kahlor LA. Effects of COVID-19 Misinformation on Information Seeking, Avoidance, and Processing: A Multicountry Comparative Study. Sci Commun. 2020;42(5):586-615. doi:10.1177/1075547020959670

15. Pelletan J. Risk perception with imperfect information and social interactions: Understanding group polarization. Bulletin of Economic Research 2021;73(4):688-703.

16. Pettersson R. Information Design–Principles and Guidelines, Journal of Visual Literacy, 2010;29:2, 167-182, DOI: 10.1080/23796529.2010.11674679.

17. Bagnoli F, Liò P, Sguanci L. Risk perception in epidemic modeling. Phys Rev E Stat Nonlin Soft Matter Phys. 2007 Dec;76(6 Pt 1):061904. doi: 10.1103/PhysRevE.76.061904.

18. Kim J-N, Gil de Zúñiga H. Pseudo-Information, Media, Publics, and the Failing Marketplace of Ideas: Theory. *American Behavioral Scientist*. 2021;65(2):163-179. doi:10.1177/0002764220950606

19. Wardle C, Singerman E. Too little, too late: social media companies' failure to tackle vaccine misinformation poses a real threat. BMJ 2021; 372 :n26 doi:10.1136/bmj.n26

20. Yasir A, Hu X, Ahmad M, Rauf A, Shi J, Ali Nasir S. Modeling Impact of Word of Mouth and E-Government on Online Social Presence during COVID-19 Outbreak: A Multi-Mediation Approach. International Journal of Environmental Research and Public Health. 2020; 17(8):2954. https://doi.org/10.3390/ijerph17082954

21. Lupton, D., Lewis, S. Learning about COVID-19: a qualitative interview study of Australians' use of information sources. BMC Public Health 21, 662 (2021). https://doi.org/10.1186/s12889-021-10743-7

22. Kandel N, Chungong S, Omaar A, Xing J. Health security capacities in the context of COVID-19 outbreak: an analysis of International Health Regulations annual report data from 182 countries. Lancet. 2020;395(10229):1047-1053. doi: 10.1016/S0140-6736(20)30553-5.

23. Data Protection Commission: Data Protection and COVID-19. Accessed at: https://www.dataprotection.ie/en/dpc-guidance/blogs/data-protection-and-covid-19

24. Jennings W, Stoker G, Valgarðsson V, Devine D, Gaskell J. How trust, mistrust and distrust shape the governance of the COVID-19 crisis, Journal of European Public Policy, 2021;28:8, 1174-1196, DOI: 10.1080/13501763.2021.1942151

25. Baillergeau E, Duyvendak J. Experiential knowledge as a resource for coping with uncertainty: evidence and examples from the Netherlands, Health, Risk & Society, 2016;18:7-8, 407-426, DOI: 10.1080/13698575.2016.1269878

26. Griffin RJ, Dunwoody S, Neuwirth K. Proposed model of the relationship of risk information seeking and processing to the development of preventive behaviors. Environ Res. 1999 Feb;80(2 Pt 2):S230-S245. doi: 10.1006/enrs.1998.3940.

For peer review only

27. Loewenstein G, Mather J. Dynamic Processes in Risk Perception. Journal of Risk and Uncertainty 1990;3(2):155–175.

28. Tversky A, Kahneman D. Judgment under Uncertainty: Heuristics and Biases. Science. 1974;185(4157):1124–1131.

29. Taleb NN, Bar-Yam Y, Cirillo P. On single point forecasts for fat-tailed variables. Int J Forecast. 2020;10. doi:10.1016/j.ijforecast.2020.08.008

30. Ingram C, Downey V, Roe M, et al. COVID-19 Prevention and Control Measures in Workplace Settings: A Rapid Review and Meta-Analysis. Int J Environ Res Public Health. 2021;18(15):7847. doi:10.3390/ijerph18157847

31. Rostami A, Sepidarkish M, Leeflang MMG, Riahi SM, Nourollahpour Shiadeh M, Esfandyari S, Mokdad AH, Hotez PJ, Gasser RB. SARS-CoV-2 seroprevalence worldwide: a systematic review and meta-analysis. Clin Microbiol Infect. 2021 Mar;27(3):331-340.

32. Scheufele, D. A., and N. M. Krause. 2019. "Science Audiences, Misinformation, and Fake News." Proceedings of the National Academy of Sciences 116 (16): 7662–7669. doi:10.1073/pnas.1805871115.

33. Harmon-Jones E, Harmon-Jones C, Levy N. An Action-Based Model of Cognitive-Dissonance Processes. Current Directions in Psychological Science. 2015;24(3):184-189. doi:10.1177/0963721414566449

34. Bansal-Travers M, Hammond D, Smith P, Cummings KM. The impact of cigarette pack design, descriptors, and warning labels on risk perception in the U.S. Am J Prev Med. 2011;40(6):674-82. doi: 10.1016/j.amepre.2011.01.021.

35. Mays D, Niaura RS, Evans WD, et al. Cigarette packaging and health warnings: the impact of plain packaging and message framing on young smokers. Tobacco Control 2015;24:e87-e92.

36. Popova L, Owusu D, Jenson D, Neilands TB. Factual text and emotional pictures: overcoming a false dichotomy of cigarette warning labels [published online ahead of print, 2017 Apr 20]. Tob Control. 2017;tobaccocontrol-2016-053563. doi:10.1136/tobaccocontrol-2016-053563

37. Nan X, Iles I, Yang B, Ma Z. Public Health Messaging during the COVID-19 Pandemic and Beyond: Lessons from Communication Science. Health Communication. 2022;37(1):1-19. doi: 10.1080/10410236.2021.1994910

38. Lasswell, H. D. (1948). The structure and function of communication in society. In L. Bryson (Ed.), *The communication of ideas* (pp. 37–51). Harper and Row.

39. Kornfield R, Smith KC, Szczypka G, Vera L, Emery S. Earned media and public engagement with CDC's "Tips from Former Smokers" campaign: an analysis of online news and blog coverage. J Med Internet Res. 2015;17(1):e12. Published 2015 Jan 20. doi:10.2196/jmir.3645

For peer review only

40. Bennett P, Noble S, Johnston S, et al COVID-19 confessions: a qualitative exploration of healthcare workers experiences of working with COVID-19. BMJ

For peer review only

Figure 1 - Outline of higher-order and lower-themes on COVID-19 communication strategies

Uploaded separately as PDF as per editorial office request.

For peer review only

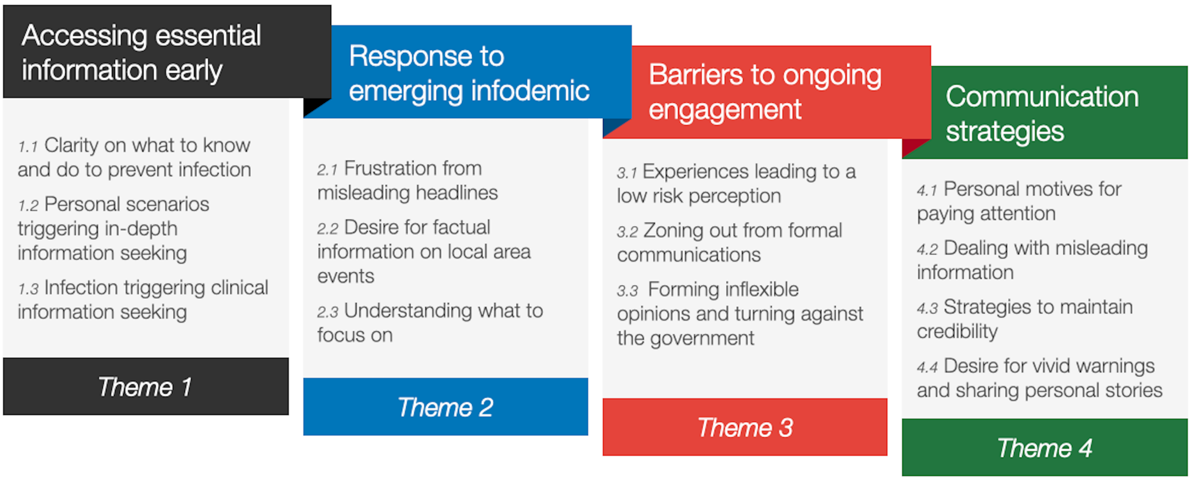

For peer review only

Standards for Reporting Qualitative Research (SRQR)*

http://www.equator-network.org/reporting-guidelines/srqr/

Title and abstract

	Page/line no(s).
**Title** - Concise description of the nature and topic of the study Identifying the study as qualitative or indicating the approach (e.g., ethnography, grounded theory) or data collection methods (e.g., interview, focus group) is recommended	1
**Abstract** - Summary of key elements of the study using the abstract format of the intended publication; typically includes background, purpose, methods, results, and conclusions	3

Introduction

**Problem formulation** - Description and significance of the problem/phenomenon studied; review of relevant theory and empirical work; problem statement	5
**Purpose or research question** - Purpose of the study and specific objectives or questions	5

Methods

**Qualitative approach and research paradigm** - Qualitative approach (e.g., ethnography, grounded theory, case study, phenomenology, narrative research) and guiding theory if appropriate; identifying the research paradigm (e.g., postpositivist, constructivist/ interpretivist) is also recommended; rationale**	7
**Researcher characteristics and reflexivity** - Researchers' characteristics that may influence the research, including personal attributes, qualifications/experience, relationship with participants, assumptions, and/or presuppositions; potential or actual interaction between researchers' characteristics and the research questions, approach, methods, results, and/or transferability	8
**Context** - Setting/site and salient contextual factors; rationale**	6
**Sampling strategy** - How and why research participants, documents, or events were selected; criteria for deciding when no further sampling was necessary (e.g., sampling saturation); rationale**	6
**Ethical issues pertaining to human subjects** - Documentation of approval by an appropriate ethics review board and participant consent, or explanation for lack thereof; other confidentiality and data security issues	2
**Data collection methods** - Types of data collected; details of data collection procedures including (as appropriate) start and stop dates of data collection and analysis, iterative process, triangulation of sources/methods, and modification of procedures in response to evolving study findings; rationale**	5

For peer review only - http://bmjopen.bmj.com/site/about/guidelines.xhtml

**Data collection instruments and technologies** - Description of instruments (e.g., interview guides, questionnaires) and devices (e.g., audio recorders) used for data collection; if/how the instrument(s) changed over the course of the study	6
**Units of study** - Number and relevant characteristics of participants, documents, or events included in the study; level of participation (could be reported in results)	7
**Data processing** - Methods for processing data prior to and during analysis, including transcription, data entry, data management and security, verification of data integrity, data coding, and anonymization/de-identification of excerpts	6
**Data analysis** - Process by which inferences, themes, etc., were identified and developed, including the researchers involved in data analysis; usually references a specific paradigm or approach; rationale**	7
**Techniques to enhance trustworthiness** - Techniques to enhance trustworthiness and credibility of data analysis (e.g., member checking, audit trail, triangulation); rationale**	7

Results/findings

**Synthesis and interpretation** - Main findings (e.g., interpretations, inferences, and themes); might include development of a theory or model, or integration with prior research or theory	8
**Links to empirical data** - Evidence (e.g., quotes, field notes, text excerpts, photographs) to substantiate analytic findings	Table 1-4

Discussion

**Integration with prior work, implications, transferability, and contribution(s) to the field -** Short summary of main findings; explanation of how findings and conclusions connect to, support, elaborate on, or challenge conclusions of earlier scholarship; discussion of scope of application/generalizability; identification of unique contribution(s) to scholarship in a discipline or field	12
**Limitations** - Trustworthiness and limitations of findings	16

Other

**Conflicts of interest** - Potential sources of influence or perceived influence on study conduct and conclusions; how these were managed	2
**Funding** - Sources of funding and other support; role of funders in data collection, interpretation, and reporting	2

*The authors created the SRQR by searching the literature to identify guidelines, reporting standards, and critical appraisal criteria for qualitative research; reviewing the reference lists of retrieved sources; and contacting experts to gain feedback. The SRQR aims to improve the transparency of all aspects of qualitative research by providing clear standards for reporting qualitative research.

For peer review only

**The rationale should briefly discuss the justification for choosing that theory, approach, method, or technique rather than other options available, the assumptions and limitations implicit in those choices, and how those choices influence study conclusions and transferability. As appropriate, the rationale for several items might be discussed together.

Reference:
O'Brien BC, Harris IB, Beckman TJ, Reed DA, Cook DA. **Standards for reporting qualitative research: a synthesis of recommendations.** *Academic Medicine*, Vol. 89, No. 9 / Sept 2014 DOI: 10.1097/ACM.0000000000000388

For peer review only

“Communication, that’s the key”: A qualitative investigation of how essential workers with COVID-19 responded to public health information

Journal:	*BMJ Open*
Manuscript ID	bmjopen-2022-061583.R2
Article Type:	Original research
Date Submitted by the Author:	09-Jun-2022
Complete List of Authors:	Roe, Mark; University College Dublin<br>Buggy, Conor; University College Dublin<br>Ingram, Carolyn; University College Dublin<br>Codd, Mary; University College Dublin<br>Buckley, Claire; Health Service Executive<br>Archibald, Mary; University College Dublin<br>Rachwal, Natalie; University College Dublin<br>Downey, Vicky; University College Dublin<br>Chen, Yanbing; University College Dublin<br>Sripaiboonkji, Penpatra; University College Dublin<br>Drummond, Anne; University College Dublin<br>Alvarez, Elizabeth; McMaster University, Health Research Methods, Evidence and Impact; McMaster University, Centre for Health Economics and Policy Analysis (CHEPA)<br>Perrotta, Carla; University College Dublin, School of Public Health, Physiotherapy and Sport Sciences
**Primary Subject Heading**:	Public health
Secondary Subject Heading:	Infectious diseases, Health policy, Communication
Keywords:	COVID-19, Public health < INFECTIOUS DISEASES, Health policy < HEALTH SERVICES ADMINISTRATION & MANAGEMENT

SCHOLARONE™
Manuscripts

For peer review only - http://bmjopen.bmj.com/site/about/guidelines.xhtml

I, the Submitting Author has the right to grant and does grant on behalf of all authors of the Work (as defined in the below author licence), an exclusive licence and/or a non-exclusive licence for contributions from authors who are: i) UK Crown employees; ii) where BMJ has agreed a CC-BY licence shall apply, and/or iii) in accordance with the terms applicable for US Federal Government officers or employees acting as part of their official duties; on a worldwide, perpetual, irrevocable, royalty-free basis to BMJ Publishing Group Ltd ("BMJ") its licensees and where the relevant Journal is co-owned by BMJ to the co-owners of the Journal, to publish the Work in this journal and any other BMJ products and to exploit all rights, as set out in our licence.

The Submitting Author accepts and understands that any supply made under these terms is made by BMJ to the Submitting Author unless you are acting as an employee on behalf of your employer or a postgraduate student of an affiliated institution which is paying any applicable article publishing charge ("APC") for Open Access articles. Where the Submitting Author wishes to make the Work available on an Open Access basis (and intends to pay the relevant APC), the terms of reuse of such Open Access shall be governed by a Creative Commons licence – details of these licences and which Creative Commons licence will apply to this Work are set out in our licence referred to above.

Other than as permitted in any relevant BMJ Author's Self Archiving Policies, I confirm this Work has not been accepted for publication elsewhere, is not being considered for publication elsewhere and does not duplicate material already published. I confirm all authors consent to publication of this Work and authorise the granting of this licence.

For peer review only

"Communication, that's the key": A qualitative investigation of how essential workers with COVID-19 responded to public health information

Mark Roe[1], Conor Buggy[1], Carolyn Ingram[1], Mary Codd[1], Claire Buckley[2], Mary Archibald[1], Natalie Rachwal[1], Vicky Downey[1], Yanbing Chen[1], Penpatra Sripaiboonkji[1], Anne Drummond[1], Elizabeth Alvarez[3], Carla Perrotta[1]

[1] School of Public Health, Physiotherapy and Sports Science, University College Dublin, Ireland

[2] Health Service Executive, Dublin, Ireland

[3] Department of Health Research Methods, Evidence and Impact, McMaster University, Canada

Corresponding author
Mark Roe
School of Public Health, Physiotherapy, and Sports Science
Health Science Building
University College Dublin
Dublin
D04 V1W8
Ireland

Email: mark.roe@ucd.ie
Phone: +353 1 716 7777

Word count: 4,485

Tables: 4

Figures: 1

For peer review only

Patient and public involvement

Patients and/or the public were not involved in the design, or conduct, or reporting, or dissemination plans of this research.

Patient consent for publication

Not required.

Ethical approval

Ethical approval was granted by the UCD Human Research and Ethics Committee (ID: LS-E-21-85-Roe-Perrotta). Verbal informed consent was obtained from the study participants.

Provenance and peer review

Not commissioned; externally peer reviewed.

For peer review only

ABSTRACT

Objectives

To understand how essential workers with confirmed infections responded to information on COVID-19.

Design

Qualitative analysis of semi-structured interviews conducted in collaboration with the national contact tracing management programme in Ireland.

Setting

Semi-structured interviews conducted via telephone and Zoom Meetings.

Participants

18 people in Ireland with laboratory confirmed SARS-CoV-2 infections using real-time polymerase chain reaction testing of oropharyngeal and nasopharyngeal swabs. All individuals were identified as part of workplace outbreaks defined as ≥2 individuals with epidemiologically linked infections.

Results

A total of four high-order themes were identified: (1) Accessing essential information early, (2) responses to emerging "infodemic", (3) barriers to ongoing engagement, and (4) communication strategies. Thirteen lower-order, or subthemes, were identified and agreed upon by the researchers.

Conclusions

Our findings provide insights into how people infected with COVID-19 sought and processed related health information throughout the pandemic. We describe strategies used to navigate excessive and incomplete information and how perceptions of information providers evolves overtime. These results can inform future communication strategies on COVID-19.

For peer review only

STRENGTHS AND LIMITATIONS

- The qualitative design enabled the collection of data on the experiences and perceptions of people who had laboratory confirmed COVID-19 infections.
- Throughout the study the researchers used several best-practice strategies to complete thematic analysis of data from semi-structured interviews.
- Given the lack of prior qualitative research on this topic and cohort, we used inductive reasoning approach which enables us to avoid assumptions of prior research or frameworks.
- Participants were selected from a sub-group of the population known as 'essential workers' that could not avail of 'remote working' throughout the pandemic making their views are critical to informing public health policies that cater for cohorts at increased risk of infection.
- Our recruitment strategy had a low response rate perhaps indicating a lack of willingness in this cohort to engage with COVID-19 research or initiatives associated with official public health organisations.

For peer review only

BACKGROUND

Sharing public health information is a crucial step in creating awareness of threats and protective strategies.[1] The COVID-19 pandemic is unique in two ways. First, it is the first pandemic where digital technologies and social media platforms are used to share information to keep people safe.[2] Second, although little information was available on the novel SARS-CoV-2 pathogen in January 2020, an ‘infodemic’ had emerged within four months, prompting global responses such as the World Health Organization’s Information Network for Epidemics (EPI-WIN).[3]

While acknowledging of wide-spread access to information on COVID-19, little is known about how people receive and process such content. This is especially true for groups at a higher risk of infection who are associated with super-spreading events (SSEs) which have a greater influence on the trajectory of epidemics.[4]

Prior research on COVID-19 information has explored the public’s perceptions of communication strategies, and their ability to correctly answer questions on COVID-19 epidemiology.[5, 6] These offer little insight into how people who were most at risk of infection (e.g. high risk workers) make sense of information on COVID-19 or how they believe communication strategies could be improved in the future.

Using semi-structured interviews, this study aims to investigate how essential workers with confirmed infections responded to information on COVID-19. This research could inform workplace-related public health communications as the current pandemic continues and future pandemics emerge.

METHODS

This study represents a sub-analysis of a preregistered study to investigate the behaviours and contextual factors associated with COVID-19 outbreaks and SSEs (OSF: https://osf.io/aeg74).

Patient and public involvement

There was no patient or public involvement in setting the research agenda.

For peer review only

Data collection methods

Semi-structured interviews were conducted using a password-protected Zoom account via a secure login. Only the researcher and invited participant were admitted to the call. A transcript of the audio recording was generated and corrected following a playback of the original audio file, enabling the researchers to remove any identifiable information. Interviews lasted a combined total of 7.6 h (average 25.2 ± 12.5 min) generating 45,987 words. Each participants was assigned a unique identification number (PID). After this process, recordings were deleted from all sources. Four researchers completed the interviews following a series of pilot interviews with fellow team members (NR = 8; MR = 6; MA = 2; CI = 2). All interviews were conducted from 22nd July 2021 to 7th Aug 2021.

For context, as of 22nd July 2021, Ireland reported 289,139 laboratory confirmed infections since the first case on 29th February 2020, equating to a population prevalence of 5.8%.[7] The median age of national cases was 22. Healthcare workers accounted for 10.3%. A total of 15,134 hospital admissions and 5,026 were reported. Approximately, 63.8% and 53.0% of the population had been partially or fully vaccinated against COVID-19. In the 14-days prior to data collection, Ireland confirmed 13,065 infections (prevalence = 0.3%) with 29.5% of cases associated with community transmission.[8] The median age was 23. A similar infection rate was reported until 13th Oct 2021. Ireland's 7-day test positivity rate was 5.9-7.9% throughout the study period.[7]

We developed an interview guide to explore three areas: behaviours and contextual factors leading to COVID-19 outbreaks, responses to minimise transmission, and lessons for future responses. These areas were identified following a review of the literature, consultation with national public health authorities, and initial findings from a larger COVID-19 research project by the authors (Science Foundation Ireland: 20/COV/8539). Within this we asked specific questions on information and public health messaging and the role of the media in minimising transmission and sustaining safe behaviours. Prompt questions were asked by the researchers to illicit more detailed information on these topics.

For peer review only

Study participants

We recruited 18 people in the Republic of Ireland who had laboratory-confirmed SARS-CoV-2 infections using real-time polymerase chain reaction testing of oropharyngeal and nasopharyngeal swabs. All individuals were identified as part of suspected workplace outbreaks where ≥2 individuals had epidemiologically linked infections. We targeted this group given their first-hand experience of (1) COVID-19 outbreaks in the workplace, and (2) responses to minimise transmission such as infection control measures in workplaces, engagement with test-and-trace services, and quarantining. Therefore, inclusion criteria included age ≥18 y and a laboratory confirmed infection likely linked to a workplace outbreak.

Potential participants were selected from a sub-group of the population known as 'essential workers' that could not avail of 'remote working' throughout the pandemic. Potential participants (n=167) were identified by the partner Health Service Executive (HSE) Contact Tracing Centre (CTC) as fulfilling the inclusion criteria, and during routine surveillance activities, were invited to receive a call from the research team. Overall, 104 individuals were contactable by the HSE CTC (62.3%). From this cohort, 64 (61.5%) refused to be contacted by the research team while 40 (38.5%) gave consent. We were able to contact and complete interviews with 18 people from this sample, representing 10.8% of potential participants originally identified by the HSE CTC.

Seven females (38.9%) and 11 males (61.1%) were interviewed. The median age was 35 y (min = 18; max = 57). Sectors represented included food, construction, laundry, retail, energy, healthcare, professional services, public services, and emergency services.

Data analysis

We used a thematic analysis approach to support a structured exploration of patterns in perceived behaviours and contextual factors associated with COVID-19 outbreaks experienced by the participants. As qualitative research seeks to generate knowledge grounded in human experience, thematic analysis is a trusted method that guides researchers in 'highlighting similarities and differences', 'exploring unanticipated insights' and patterns, and 'summarising key features' of data that cannot be

For peer review only

statistically analysed.[9] Failure to overlook the important of qualitative methods diminishes our understanding of how people experience specific events and how that experience might be enhanced through new considerations when designing interventions (e.g. infection control measures in high-risk workplaces).

The coders (MR x 10, VD x 8) familiarised themselves with the transcripts prior to initiating coding. Thematic analysis was conducted in accordance with the six phases of trustworthy thematic analysis as outlined by Nowell et al. (2017) (i.e. familiarisation with the data; generating initial codes; searching for themes; reviewing themes; defining and naming themes; producing the report).[9] Qualitative analysis software (NVivo V12) was used to transform raw data into multiple codes using open and axial coding processes. A final of 35 codes were computed.

We considered saturation was met when two conditions were realised: (1) agreement between researchers that no further transcript codes could be generated, and (2) no further information, deemed relevant to the stated research questions, could be obtained from the transcripts.[10] Discrepancies were resolved through discussion until a consensus was reached. Reflective discussions were conducted (MR, VD, CI) throughout the study to review the rationale for selecting codes, themes, and quotes.[11]

Credibility strategies used throughout analysis included (1) critical friend approach by encouraging reflexivity by asking provocative questions to critique our research in a supportive manner that clarifies ideas and interpretation, and (2) cross-checking of themes identified in prior focus group studies.[12] The team has a diverse range of background (e.g. medicine, public health, epidemiology, risk management, occupational health, psychology) that facilitated a robust review of identified themes. Results are reported in line with SRQR reporting guidelines (Supplementary file 1).[13]

RESULTS

A total of four high-order themes were identified: (1) Accessing essential information early, (2) responses to emerging infodemic, (3) barriers to ongoing engagement, and (4) communication strategies. Thirteen lower-order, or subthemes, were identified and agreed upon by the researchers (Figure 1).

For peer review only

Theme 1: Accessing essential information early

The first theme highlights that participant reported access to information on the essential elements of COVID-19 before a pandemic was first declared in March 2020. The identified sub-themes reveal the role of clear communications and personal thresholds for seeking information to reduce transmission of the virus in work settings.

Sub-theme 1.1: Clarity on what to know and do to prevent infection

Participants expressed the view that since COVID-19 was first confirmed in Ireland on 29th February 2020, public health information was almost unavoidable given the extent of coverage on news programmes and social media. Most participants accessed information via national television news shows which included live broadcasts of public health press briefings and newspapers as well as websites of the HSE and World Health Organization. Additionally, every participant reported having access to information on COVID-19 in their workplace. Workplace information provided clarity on what staff could expect if workers developed symptoms of COVID-19. Interestingly, it appears that prior to their infection, participants largely tended to only seek generic information on COVID-19 that was deemed necessary for functional tasks (table 1).

Sub-theme 1.2: Personal scenarios triggering in-depth information seeking

Participants referred to triggers that initiated searches to answer specific queries related to COVID-19 that arose from unique personal events (table 1). This was largely indicative of personal concerns related to changes in their own health status, or that of an acquaintance or family member, leading to a reappraisal of the threat posed by COVID-19. More in-depth information was sought from specific Google searches, YouTube videos, and formal research papers. It appears these intentional information seeking activities served the specific purpose of helping the participants make sense of their personal scenarios.

Sub-theme 1.3: Infection triggering clinical information seeking

We also noticed that even following confirmation of a positive test, participants only sought information when COVID-19 began to impact their health (table 1). A clear distinction was noted between this and sub-theme 1.2, as information seeking

For peer review only - http://bmjopen.bmj.com/site/about/guidelines.xhtml

occurred after a specific event (i.e. following confirmed infection) and refers to the specific domain of case management (i.e. what are the potential clinical outcomes, and how what actions can support better outcomes). Participants expressed that these searches for specific clinical information allowed them to prepare for what might be expected to occur throughout their period of self-isolation.

For peer review only

Table 1 – Illustrative quotes supporting theme one 'accessing essential information early'

Sub-Theme	Supporting Quotes	Participant ID
1.1 Clarity on what to know and do to prevent infection	You were kind of just told what the symptoms were, and you were educated on what symptoms to look out for and if you have any of these symptoms to report them and to isolate at home.	PID 12: Nurse
	Everyone was already pretty much informed of what would happen if there was a scenario where someone gets sick.	PID 17: Meat plant worker
	The procedures were rolled out nationally. There was no issue with dissemination of information. The quality of information coming at a national level, it was good.	PID 20: Emergency services worker
	There's a lot of access [to information] in every part of the company. [It's been] very very good through the COVID period so I give out credit for that.	PID 7: Food factory worker
	I got the information I needed, I never looked for any more information that I didn't need.	PID 12: Nurse
1.2 Personal scenarios triggering in-depth information seeking	It's [not] until either they get COVID or they see someone that they know get COVID badly, once that happens then they'll realize what COVID is, and that's the sad reality is that there's a lot of people who won't take this thing seriously until they see somebody that they know with COVID.	PID 1: Meat plant worker
	When I got the headache I obviously was looking up to see was this part of the symptoms. Is it actually COVID or is it just this cough I had before Christmas? So I definitely was seeking out symptom information, and I read, I don't know it was like a dissertation or a there was a paper written on this 72 hour headache that wouldn't react to any medication or anything so that kind of rang true.	PID 16: Office worker in professional services
1.3 Infection triggering clinical information seeking	I won't really be looking for information unless I like start showing symptoms and I start to feel very unwell then I'll start looking at it going like, right, what is it, how can I help myself with this, what can I do.	PID 12: Nurse
	[At the time] I was just kind of reading through different information about symptoms and how long they last and different things that can happen.	PID 16: Office worker in professional services

For peer review only

Theme 2: Response to emerging infodemic

The second theme emerged in response to participants frequently discussing the rapid growth of information on COVID-19 despite it often being incomplete, and at times, misleading or of little use to inform their personal actions. Here we noticed a desire for information based on how the COVID-19 epidemic was progressing in local areas.

Sub-theme 2.1: Frustration with misleading headlines

We repeatedly found references to perceived over-reporting on COVID-19 by the media (table 2). Whilst participants mentioned that although the sustained media coverage was at times fear-provoking, it also played a useful role in keeping the public informed of the latest COVID-19 trajectory. Participants reported being disheartened by the manner of reporting, as opposed to the constant publication of media content related to COVID-19. Similarly, participants also alluded to their distaste for some marketing tactics, deployed by some media outlets to increase online traffic, which had the potential to misinform the public.

Sub-theme 2.2: Desire for factual information on local area events

Our analysis identified that participants valued information related to their local geographical area, going beyond the reporting of mere confirmed case numbers to reveal details of infection sources and clinical outcomes (table 2). This was seen as beneficial to raising awareness of the threat in the area in attempts to bolster adherence to infection control measures.

Sub-theme 2.3: Understanding what to focus on

Despite repeated references to excessive amounts of information on COVID-19 in the media and online, participants appear to have coped by considering the relevance of the content for understanding the nature of the virus, and how it could inform their future actions (table 2).

For peer review only

Table 2 – Illustrative quotes supporting theme two 'response to emerging infodemic'

Sub-Theme	Supporting Quotes	Participant ID
2.1 Frustration with misleading headlines	I feel like the cases are kind of annoying hearing about [them] every day, but I feel like it is good to know… I think they just report on it too often kind of scaring people like as in they kind of use headlines to scare people, when you read the actual information it's very misleading.	PID 14: Factory worker
	Give proper factual information instead of kind of click bait headlines on everything.	PID 14: Factory worker
2.2 Desire for factual information on local area events	There should be maybe more information on it like how many people are like in hospital in each area and like where the cases are explicitly coming from. [I] think it'd be more beneficial than maybe just hearing the cases every day, you kind of stop listening and stop caring, there's like 1,000 cases when like 4 million people like it doesn't, but if you kind of read it and know people are near you it might make people pay more attention and stick to the rules and keep safe	PID 14: Factory worker
	I think the government needs to be more transparent on things and provide more accurate information because at the time when this whole pandemic was rampant I feel like the government was just overloading people with so much information that you didn't even know which one is right which one is wrong. You could be saying 'oh that's what I heard is' and then another person could be saying 'oh no, this is what I heard is' and like you know it's all a bunch of everything. All the information is all jumbled up and the truth is somewhere in the middle.	PID 17: Meat plant worker
2.3 Understanding what to focus on	It's just knowing what this virus is and knowing how it's going to affect your body and being able to prepare yourself for when it's going to affect your body	PID 1: Meat plant worker
	I was just reading more about it and what it actually does and how or what I'm supposed to take or do to make my immune system stronger, so those were the things I was checking	PID 3: Laundry worker
	I guess the main thing is have information readily available for everybody just to keep on track and keep updated on what the most recent guidelines are.	PID 12: Nurse

For peer review only

Theme 3: Barriers to ongoing engagement

Theme three may be best conceptualised as the consequences of participants having to seek and process information on COVID-19 in a largely ad-hoc personally motivated manner (theme 1) whilst dealing with the factors of incomplete and context-free information (theme 2). We heard how this led some essential workers to reduce their perception in the threat of COVID-19 whilst simultaneously changing the nature of their engagement with official sources of public health information. Within this process, social media sites became platforms playing an ever-increasing role in re-forming opinions of COVID-19 as well as the government and officials responding to it. This was rooted in a perceived mismatch between information reported on COVID-19 and insights gained from first-hand experience of being infected, resulting in some participants stating that they will be less likely to engage with communications in the future.

Sub-theme 3.1: Experiences leading to a low-risk perception

We noted that several participants, having reflected on how the congruency between their personal experiences throughout the pandemic and those reported by the media, expressed a current belief that COVID-19 was not as dangerous as reported in the media (table 3). These participants believed that they correctly understood the content of prior media reports, yet witnessed, or heard about, contradictory COVID-19 outcomes prior to adopting their alternative beliefs. A source of evidence for personal experiences leading to a lower risk perception appears to be perceived wrong information that was reported yet never acknowledged or corrected. We noted that such participants were eager to emphasise that they were not denying the existence of COVID-19.

Of 18 participants, we encountered only one that alluded to conspiracy theories. Thus, we draw a clear distinction between the emergence of alternative hypothesises, at odds with the narratives reported by official authorities or media outlets, and conspiracy theories citing malicious motives by the official organisations.

Sub-theme 3.2: Zoning out from formal communications

Participants referred to a reduced ability to continually engage with media and public health messages over time, resulting in unintended outcomes such as when some people become “accustomed to hearing about it and stop caring”. Formal communications strategies (i.e. content released by national health authorities such as press briefings) on COVID-19 in Ireland were first implemented in February 2020. We noted the belief that the public developed a reduced willingness, or capacity, to engage in this form of messaging as the pandemic progressed (table 3). Instead, participants mentioned the role of ‘informal advertisements’, associated with the placing of public health messages during the start and end of popular programs and delivered by the host.

Sub-theme 3.3: Forming inflexible opinions and turning against the government

Participants recognised the role of social networks, particularly those online, in shaping peoples’ interpretation of information related to COVID-19. Specifically, we noted the perception that online social networks were effective at facilitating a process whereby users arrived at an interpretation of COVID-19 information that was more likely to be anti-establishment (table 3).

Participants spoke of the consequences of prior measures taken to reduce transmission of COVID-19. In particular, national lockdowns, known locally as ‘Level Five restrictions’ were identified as being a source of anger amongst the public. We found that participants were unlikely to dissociate government officials from senior members of Ireland’s National Public Health Emergency Team. Additionally, there was a sentiment that the public’s negative reaction to previous COVID-19 responses would lead some people to adopt a negative attitude of future messages regardless of the content (table 3).

For peer review only

Table 3 – Illustrative quotes supporting theme three 'barriers to ongoing engagement'

Sub-Theme	Supporting Quotes	Participant ID
3.1 Experiences leading to a low risk perception	I believe soon it's coming, another lockdown. Soon, very soon I believe, and I think that you know that already. There are so many cases that's why. Because I said like I don't know how [they're] can be so many cases. But we never... heard, like we saw on the news there are few cases like 900 or something like that a day... but we didn't hear like somebody had for real COVID.	PID 22: Meat plant worker
	It's not like we don't believe about COVID, alright it's a virus I understand, but it's not as worse [as] they actually say… How can you believe everything they say on the news and everything when people every day see different?	PID 21: Construction worker
3.2 Zoning out from formal communications	So the only information I really looked for was [when symptomatic], I was talking to my boss at the time so he was the one who was keeping me informed with what I should be doing at all, so uh he told me to stay off. I think it was 2 days and then if I still had symptoms or if symptoms [hadn't] got better I was to get a test at the GP.	PID 17: Meat plant worker
	I feel like some people when they kind of read the news like what will make them be more careful, but sometimes it's the opposite. Like if people are hearing it every day they just kind of just become accustomed to hearing about it and stop caring.	PID 13: Nurse
	You always hear [TV host] when he's on [Ireland's most popular entertainment show] wrapping it up saying "everyone make sure to wear your masks and wash your hands", so if it was kind of embedded into more of an informal advertisement. I think it could be responded to better or even like on the radio channels just that it's nearly embedded subconsciously into people. But I think the formality on how they're doing it now, it's just lost the interest of people. People are just zoned out they don't care anymore about it.	PID 13: Nurse
3.3 Forming inflexible opinions and turning against the government	People are very headstrong. They'll keep this thought that they have of whatever their opinion on COVID is because, like, they'll have one or two people agree with them on Facebook or something, and they'll think alright well what I'm saying is correct.	PID 1: Meat plant worker
	Some people like they read too much online on Facebook, on whatever like Instagram or whatever. And then [get] sucked into it and then they end up believing it, and then it just keeps going on and on until they're sitting there telling people that COVID isn't real or that the vaccine is trying to control them. But, like, it's just that some people think that anything they're told shouldn't be taken at face value. Some people find it hard that when the government says something, that's the truth, some people do find that very hard.	PID 1: Meat plant worker
	The main thing is, especially with the measures that we had in place when we were in Level Five, I think those were very, they were very harsh. I understand. Yet it did work. But it didn't really. Like, there were a lot of people that turned against the government.	PID 1: Meat plant worker
	I think to be honest with ya a lot of people have a negative response to the government and most of the directors I've heard from the government. I know a lot of people don't like [Ireland's Chief Medical Officer]. I don't mind him, but a lot of people don't like him, and if they say something they're [of] the attitude that they're not just going to take it on board, they're just going to be disrespectful to him and it's just going to ignored from the get go.	PID 13: Nurse

For peer review only

Theme 4: Communication strategies

The final theme we identified related to how participants believed that future communication strategies on COVID-19 and future public health emergencies could be improved.

Sub-theme 4.1: Personal motives for paying attention

Despite referring to excessive information and media coverage, all participants appeared to value ongoing communication. Similar to our findings in sub-theme 1.2 (i.e. personal scenarios triggering in-depth information seeking), participants expressed a need for future communications to be anchored to a personal value proposition, such as protecting loved ones (table 4).

Sub-theme 4.2: Dealing with misleading information

All participants used social media yet were unclear about specific measures that could be taken to reduce the misinformation on digital platforms (table 4).

Sub-theme 4.3: Strategies to maintain credibility

Our analysis indicates that to avoid misinformation, participants adopted a strategy of seeking information from several recognised health authorities, rather than a sole source, before cross-checking the content with media reports (table 4).

Sub-theme 4.4: Desire for vivid warnings and sharing personal stories

When considering lessons for future strategies to improve adherence to public health measures, participants again referred to personal scenarios. Specifically, strategies used to respond to promote awareness of other public health threats, such as smoking, were mentioned by participants (table 4).

For peer review only

Table 4 – Illustrative quotes supporting theme four ‘communication strategies’

Sub-Theme	Supporting Quotes	Participant ID
4.1 Personal motives for paying attention	Communication that’s the key to explain to them, to explain things to them because if you love someone, your parent, if your parent is vulnerable, you need to protect them, we protect each other… if I protect myself, I protect also my family… If we pay attention [to] the information, we can protect each other.	PID 4: Laundry worker
	The information being disseminated is continued and is of good quality but that depends on the individual person, whether they decide to look at that information or read it. There was no onus on anybody to read it and if they didn’t it wouldn’t make a whole lot of difference to be honest with ya.	PID 20: Emergency services worker
	I think some people, they should get more information when we know [that COVID-19] is really happening in the world. So me and my daughter and my family we got [the] most information we could. But all the time we leave we put mask on, alcohol gel we have in handbag, all the time using sanitizer to clean the doors and stairs and everything. But when I start work, I see some people they doesn’t care so much, you know.	PID 7: Food processing plant general manager
	I mean one key piece of information that I’d say is make sure people know that in the case of testing positive they won’t go without pay. Because like I can see that causing a lot of unrest with people because you know it’s like I said, a lot of people have families to feed you know and people rely on them for the money to get by. If people knew that and it’s like 'oh I’ll be taken care of if this happens' you know, they’ll be a lot more willing to cooperate with the normal guidelines.	PID 17: Meat plant worker
4.2 Dealing with misleading information	Maybe if there was something in place to kind of stop all the false news and kind of stuff that’s spread online.	PID 14: Factory worker
	[When tested positive] the HSE [national health service] send me email for ten days about what I do [and to ask] how I feel.	PID 9: Meatplant worker
4.3 Strategies to maintain credibility	All the guys are doing everything that [they] can do because there’s so much more information about this... Because everything we know we get it from HSE [Health Service Executive], we get it from news, we get it from TV. Like everything, we get it from information from the health organisations giving to us.	PID 7: Food processing plant general operations
	It's just about reinforcing the information, the guidelines. It's a bit like any other training that we do in healthcare when it comes to basic life support or infection control or anything like that. We do annual updates of that every year and that is the simple purpose to keep the practice up, to keep your skills up and to keep it on the forefront of your mind so that you don’t kinda forget and let standards slip. I think that’s really the best way really to go forward, is to have support if you have any questions and have that support readily available but as well as that just having the information there and having regular updates and what’s best to do.	PID 12: Nurse, female
	[When infected] I had access from the [contact tracing caller] to all the information. I feel like they had all the information.	PID 8: Meatplant worker

4.4 Desire for vivid warnings and sharing personal stories	They should have a picture of what COVID does to your body and they'll help people realize because, like, on the smoking packets, it does help people realise and they say on them like the dangers of smoking. It shows the harsh reality of it, and that's what people need to realise is the harsh reality.	PID 1: Meat plant worker
	From a public point of view, all I can say is again information. Just that information is put out there readily for everyone. That it's clear, it's concise, it's honest, and it's blunt with regards to the impact that it will have and what's expected.	PID 12: Nurse
	Like I got infected. I could tell them, 'this is my story and yes it [is] real. You should believe this'.	PID 3: Laundry worker
	Thank you for calling and thank you for letting me explain my experience because it's very hard when you are alone. Like I'm single mother, just me and my daughter, so if something happen with me I was scared with who was going to look after my baby and all this things. But at same time [being infected] make me more strong, make me look ahead and say listen 'this is going to pass, this is not going to affect you forever, this is just a moment, uh, so keep calm'.	PID 7: Food processing plant general operations

For peer review only

DISCUSSION

Our study investigated how essential workers with confirmed infections responded to public health information on COVID-19. Analysis of semi-structured interviews with 18 participants identified four themes: (1) accessing essential information early, (2) response to emerging infodemic, (3) barriers to ongoing engagement, and (4) communication strategies. No hypothesis was tested. Instead, we used inductive reasoning to explore patterns in COVID-19 information usage, identifying several findings that can inform public health information strategies.

The precursor for seeking information is a perceived need to understand (i.e. describe what is) and respond (i.e. prescribe what should be done) to an unfolding event (i.e. COVID-19).[14] These needs evolve overtime, are directed by personal experiences, and determine the perceived importance of seeking the best possible information or making correct decisions.[15]

Given the extent of coverage in the media, information on COVID-19 was practically unavoidable in 2020. Early information on COVID-19 appears to have had the effect of focusing attention on symptoms and how suspected cases should be managed. These early 'fundamental truths', even amid incomplete information, were simple to process and capable of informing public behaviours.[16] This is important as most of the public in the region of study has had no prior experience of pandemic pathogens.

We identified that most participants obtained information from official organisations such as national and international health organisations, indicating that the people interviewed in the current study saw these sources as credible, or at least, worth seeking guidance from. In some instances, failure to share the best available information led people to seek info from misleading sources as described in subthemes 2.2 *desire for factual information on local area events,* and 3.2 *zoning out from formal communications*. Disengagement is happening and can be problematic. Yet as it happens, we see examples of lived experiences contributing to healthy behaviours. Thus, a solution might be to facilitate exchanges between producers of information and the general population to (1) ensure that needs and population expertise are being heard and (2) encourage engagement by building trust through dialogue and providing ongoing explanation.

For peer review only

Prior studies provide evidence that the perceived risk of infection depends on the proportion of neighbours that are ill, meaning that amid a lack of official communication on local events, people turn to personal networks to gain information on their neighbourhood.[17] In the era of digital networks, this inevitably funnels people towards social media platform, increasing exposure to misinformation and polarising opinions.[18]

We note calls for social media companies to regulate information deemed inaccurate, false, or malicious.[19] However, that is unlikely to satisfy information needs as people will most likely default to word of mouth.[20] For instance, Lupton and Lewis (2021) noted that the public use informal communications with friends and families, local and internationally, to keep up to date with emerging information.[21]

Whilst the role of clickbait headlines and social media is undoubtedly playing a role in spreading misinformation, so too is the inability of public health authorities to collect and disseminate information to people on live outbreaks in their communities. Surveillance capacities are a key component of the International Health Regulations for preparing and responding to infectious disease threats.[22] Tools such as community outbreak alerts might be particularly useful, especially as attention to specific diseases may decline after surges of infection when relatively low national figures are communicated.[17]

Although tools such as community outbreak alerts are common in many jurisdictions, Irish health authorities have not used them citing concerns related to data privacy, however, guidance from the national Data Protection Commissioner on 6th Mar 2020 states that “Article 9(2)(i) of [General Data Protection Regulations] GDPR and Section 53 of the Data Protection Act 2018 will permit the processing of personal data, including health data, once suitable safeguards are implemented. Such safeguards may include limitation on access to the data, strict time limits for erasure, and other measures such as adequate staff training to protect the data protection rights of individuals”.[23]

It is foreseeable that people receiving information at odds with their personal experiences creates doubts in credibility of sender. Our results imply that overtime, participants adopted a cautious attitude or scepticism towards information shared by

official and government organisations, indicating a degree of frustration and mistrust.[24]

Gaps in information lead to more reliance on experiential knowledge, a recognised useful resource when responding to uncertainty (e.g. learning from community leaders or professional mentors) especially if derived in settings that are difficult for outsiders to grasp.[25, 26] This is likely attributable to concerns about COVID-19, and the response to it, 'bubbling up' slowly overtime as the pandemic progresses.[27] Factors contributing to these changes in personal risk perceptions included delays in collecting and reporting information, the presence of random noise in that information, and the shared belief in social networks that the posed risk is reducing over time.[28] Hence, we observed an interplay between people making an effort to stay informed whilst simultaneously withdrawing disengaging from previously trusted sources, leading to emergence of more settled, and potentially alternative, beliefs running counter to official interpretations.[24] We believe this is also evident in our ability to only reach 10.8% of our identified sample.

Adopting beliefs by processing imperfect information through social interactions indicates the use of well-documented heuristic principles, mainly the representativeness heuristic (i.e. comparing risk situations with those that seem similar), the availability heuristic (i.e. assessing risk with information most easily accessed), and the anchoring heuristic (i.e. risk estimate based on previous event).[15, 28] Whilst this may seem undesirable to evidence-based domains, heuristics and risk management principles continue to form the basis of many effective COVID-19 measures that are defendable even in the absence of scientific evidence.[29, 30]

Additionally, the true risk of COVID-19 events has been difficult to accurately assess and provide information on. For example, as evidenced by large differences between reported case numbers and seroprevalence studies.[31] Overtime, as dissatisfaction manifests and trust in the information sender is eroded, it is unlikely that responses such fact-checking will deliver an immediate change in perception or behaviour.[32] In line with the action-based model of cognitive-dissonance processes, the sensation leading to a "zoning out", signalling a distancing from pre-trusted sources, as

For peer review only

inconsistencies in information and cognition must be resolved through alternatives means for the person to take logical course of action.[33]

Hence, it is oversimplistic to label 'zoning out' as a symptom of a catch-all term like pandemic fatigue (i.e. a perceived exhaustion of cognitive and emotional capacities to sustain the effort required to positively respond to outbreaks), given that it is influenced by a complex two-way sender-receiver process of detecting, disseminating, and deciphering key data, including outcomes of policies adopted by public health officials and government. Instead, it is perhaps more accurate to consider 'zoning out' as a stage in response to incomplete information from formal releases from state agencies that leads people to move away from previously reliable sources to seek additional information.

Participants referenced previous public health campaigns that involved graphic health warnings on cigarette packaging. Research has shown that the public have a preference for larger, pictorial, and loss-framed warning labels given that these are more likely to attract attention and further thought or information seeking on health risks.[34] Such communication strategies have been shown to be particularly effective at generating motivation to change behaviour in cohorts aged 13-30 y; a group more likely to online social media.[35, 36]

Ultimately, this emphasises the importance of message content (i.e. what to say) and message executions (i.e. how to say it) when communicating information on COVID-19.[37]. The need for reiterative approaches to develop public health communications has been highlighted and largely involves monitoring perceptions of message receivers of classic message inputs such as who, says what, through which channel, to whom, and with what effect.[37, 38] However, regardless of the message executions or framing, our study found that interviewed participants were sceptical about the underlying assumptions of message content (e.g. accuracy of information being communicated, or the relevance to their local community).

We also note similarities between our findings on communication strategies and those from a thematic analysis of social media posts linked to a video-based quit smoking campaign that exposed the public to comments deemed both oppositional (e.g. role of government, bigger perceived threats, scientific scepticism) and supportive (e.g.

For peer review only

personal stories, support for change, reactions to ad content).[39] Critics of graphic health warnings often believe that these strategies are emotional and not factual, however, evidence suggests that there is little difference in emotional response to information presented in graphic or factual text form, suggesting a false dichotomy.[36]

Whilst sustained communication can overwhelm, it is also a valued strategy to alleviate anxieties and uncertainty. For messages to provide clarity they must recognise both the change in information and misinformation.[21] This is because the public recognise that COVID-19 misinformation is common and expect scientists and governments to provide accurate information.[21]

Given the ever-evolving nature of infectious pathogens, the rapid detection and dissemination of health threats are heavily reliant on IT infrastructure for two-way communication between health providers and the public.[22, 24]

We also note references to sharing stories by participants. Like Bennett et al., we believe that future communication strategies could learn from personal experiences of people infected with COVID-19 if given “an outlet [to hear their] uncensored stories”.[40]

STRENGTHS AND LIMITATIONS

Our novel study design involved a partnership with the national contact tracing management programme. The qualitative design enabled the collection of data on the experiences and perceptions of people who had laboratory confirmed COVID-19 infections. Throughout the study the researchers used several best-practice strategies to complete thematic analysis of data from semi-structured interviews. Additionally, given the lack of prior qualitative research on this topic and cohort, we used inductive reasoning approach which enables us to avoid assumptions of prior research or frameworks.

A strength of our approach is the focus on essential workers given their repeated exposure to higher-risk settings throughout the pandemic, particularly when stay at home orders were imposed for the rest of society. This allows us to capture unique experiences of essential workers who were at the coalface of dealing with the threat of COVID-19 before widespread vaccination in society. It is likely that these shaped

participants perceptions of national responses to COVID-19 as outlined in theme three.

A potential limitation of this study is that our recruitment strategy had a low response rate perhaps indicating a low willingness in this cohort to engage with COVID-19 research or initiatives associated with official public health organisations. This may bias our sample and could be addressed through future contact tracing strategies. Additionally, our sample size (n=18) means our results cannot be generalised to the population or workforce as a whole. However, as participants represented essential workers, awareness of their views are important to inform public health policies that cater for cohorts at increased risk of infection.

For peer review only

Contributorship Statement

MR, CI, MA, NR, CP, MC, CB, CB, YC, PS, AD, and EA conceptualised and designed the study, and reviewed and revised the manuscript. MR, CI, MA, NR, CP, MC, CB, and CB designed the data collection instruments and recruited participants. MR, CI, MA, and NR conducted qualitative interviews. MR, VD, and CI analysed qualitative data. MR and CI drafted the initial manuscript. All authors approved the final manuscript as submitted and agree to be accountable for all aspects of the work.

Competing interests

None declared.

Data availability statement

Data are available on reasonable request. Raw transcripts of the data analysed in the study are available from the lead author.

Funding

This work was supported by Science Foundation Ireland (SFI) through the COVID-19 Rapid Response Research and Innovation Programme, grant number 20/COV/8539.

For peer review only

References

1. Mheidly N, Fares J. Leveraging media and health communication strategies to overcome the COVID-19 infodemic. J Public Health Policy. 2020;41(4):410-420. doi: 10.1057/s41271-020-00247-w.

2. Fagherazzi G, Goetzinger C, Rashid MA, Aguayo GA, Huiart L. Digital Health Strategies to Fight COVID-19 Worldwide: Challenges, Recommendations, and a Call for Papers. J Med Internet Res. 2020;22(6):e19284. doi: 10.2196/19284.

3. World Health Organization: About EPI-WIN. Accessed at: https://www.who.int/teams/risk-communication/about-epi-win

4. Broomell S, Kane P. Perceiving a pandemic: Global–local incompatibility and COVID-19 superspreading events. Decision, 2021;8(4), 227–236. https://doi.org/10.1037/dec0000155

5. Malecki KMC, Keating JA, Safdar N. Crisis Communication and Public Perception of COVID-19 Risk in the Era of Social Media. Clin Infect Dis. 2021;72(4):697-702. doi:10.1093/cid/ciaa758

6. Jaber RM, Mafrachi B, Al-Ani A, Shkara M. Awareness and perception of COVID-19 among the general population: A Middle Eastern survey. PLoS One. 2021;16(4):e0250461. doi:10.1371/journal.pone.0250461

7. Government of Ireland and Ordnance Survey Ireland: Ireland's COVID-19 Data Hub. Accessed at: https://covid-19.geohive.ie/search

8. Health Surveillance Protection Centre: Epidemiology of COVID-19 in Ireland: HPSC interim 14 day report on cases uploaded to the COVID Care Tracker. Accessed at: https://www.hpsc.ie/a-z/respiratory/coronavirus/novelcoronavirus/surveillance/covid-1914-dayepidemiologyreports/2021/july2021/COVID-19_Interim%2014%20day%20report_INTERIM_20210722_WEBSITE.pdf

9. Nowell LS, Norris JM, White DE, Moules NJ. (2017). Thematic Analysis: Striving to Meet the Trustworthiness Criteria. International Journal of Qualitative Methods. https://doi.org/10.1177/1609406917733847

10. Fusch PI, Ness LR. Are We There Yet? Data Saturation in Qualitative Research. The Qualitative Report 2015;20(9):1408-1416. https://doi.org/10.46743/2160-3715/2015.2281

11. Ortlipp M. Keeping and using reflective journals in the qualitative research process. Qual Rep 2008;13:695–705.

12. O'Brien BC, Harris IB, Beckman TJ, Reed DA, Cook DA. Standards for reporting qualitative research: a synthesis of recommendations. Acad Med. 2014;89(9):1245-1251.

13. Smith B, McGannon K. Developing rigor in qualitative research: problems and opportunities within sport and exercise psychology. International Review of Sport and Exercise Psychology. 2018;11(1):101-121. doi: 10.1080/1750984X.2017.1317357

For peer review only

14. Kim HK, Ahn J, Atkinson L, Kahlor LA. Effects of COVID-19 Misinformation on Information Seeking, Avoidance, and Processing: A Multicountry Comparative Study. Sci Commun. 2020;42(5):586-615. doi:10.1177/1075547020959670

15. Pelletan J. Risk perception with imperfect information and social interactions: Understanding group polarization. Bulletin of Economic Research 2021;73(4):688-703.

16. Pettersson R. Information Design–Principles and Guidelines, Journal of Visual Literacy, 2010;29:2, 167-182, DOI: 10.1080/23796529.2010.11674679.

17. Bagnoli F, Liò P, Sguanci L. Risk perception in epidemic modeling. Phys Rev E Stat Nonlin Soft Matter Phys. 2007 Dec;76(6 Pt 1):061904. doi: 10.1103/PhysRevE.76.061904.

18. Kim J-N, Gil de Zúñiga H. Pseudo-Information, Media, Publics, and the Failing Marketplace of Ideas: Theory. *American Behavioral Scientist*. 2021;65(2):163-179. doi:10.1177/0002764220950606

19. Wardle C, Singerman E. Too little, too late: social media companies' failure to tackle vaccine misinformation poses a real threat. BMJ 2021; 372 :n26 doi:10.1136/bmj.n26

20. Yasir A, Hu X, Ahmad M, Rauf A, Shi J, Ali Nasir S. Modeling Impact of Word of Mouth and E-Government on Online Social Presence during COVID-19 Outbreak: A Multi-Mediation Approach. International Journal of Environmental Research and Public Health. 2020; 17(8):2954. https://doi.org/10.3390/ijerph17082954

21. Lupton, D., Lewis, S. Learning about COVID-19: a qualitative interview study of Australians' use of information sources. BMC Public Health 21, 662 (2021). https://doi.org/10.1186/s12889-021-10743-7

22. Kandel N, Chungong S, Omaar A, Xing J. Health security capacities in the context of COVID-19 outbreak: an analysis of International Health Regulations annual report data from 182 countries. Lancet. 2020;395(10229):1047-1053. doi: 10.1016/S0140-6736(20)30553-5.

23. Data Protection Commission: Data Protection and COVID-19. Accessed at: https://www.dataprotection.ie/en/dpc-guidance/blogs/data-protection-and-covid-19

24. Jennings W, Stoker G, Valgarðsson V, Devine D, Gaskell J. How trust, mistrust and distrust shape the governance of the COVID-19 crisis, Journal of European Public Policy, 2021;28:8, 1174-1196, DOI: 10.1080/13501763.2021.1942151

25. Baillergeau E, Duyvendak J. Experiential knowledge as a resource for coping with uncertainty: evidence and examples from the Netherlands, Health, Risk & Society, 2016;18:7-8, 407-426, DOI: 10.1080/13698575.2016.1269878

26. Griffin RJ, Dunwoody S, Neuwirth K. Proposed model of the relationship of risk information seeking and processing to the development of preventive behaviors. Environ Res. 1999 Feb;80(2 Pt 2):S230-S245. doi: 10.1006/enrs.1998.3940.

For peer review only

27. Loewenstein G, Mather J. Dynamic Processes in Risk Perception. Journal of Risk and Uncertainty 1990;3(2):155–175.

28. Tversky A, Kahneman D. Judgment under Uncertainty: Heuristics and Biases. Science. 1974;185(4157):1124–1131.

29. Taleb NN, Bar-Yam Y, Cirillo P. On single point forecasts for fat-tailed variables. Int J Forecast. 2020;10. doi:10.1016/j.ijforecast.2020.08.008

30. Ingram C, Downey V, Roe M, et al. COVID-19 Prevention and Control Measures in Workplace Settings: A Rapid Review and Meta-Analysis. Int J Environ Res Public Health. 2021;18(15):7847. doi:10.3390/ijerph18157847

31. Rostami A, Sepidarkish M, Leeflang MMG, Riahi SM, Nourollahpour Shiadeh M, Esfandyari S, Mokdad AH, Hotez PJ, Gasser RB. SARS-CoV-2 seroprevalence worldwide: a systematic review and meta-analysis. Clin Microbiol Infect. 2021 Mar;27(3):331-340.

32. Scheufele, D. A., and N. M. Krause. 2019. "Science Audiences, Misinformation, and Fake News." Proceedings of the National Academy of Sciences 116 (16): 7662–7669. doi:10.1073/pnas.1805871115.

33. Harmon-Jones E, Harmon-Jones C, Levy N. An Action-Based Model of Cognitive-Dissonance Processes. Current Directions in Psychological Science. 2015;24(3):184-189. doi:10.1177/0963721414566449

34. Bansal-Travers M, Hammond D, Smith P, Cummings KM. The impact of cigarette pack design, descriptors, and warning labels on risk perception in the U.S. Am J Prev Med. 2011;40(6):674-82. doi: 10.1016/j.amepre.2011.01.021.

35. Mays D, Niaura RS, Evans WD, et al. Cigarette packaging and health warnings: the impact of plain packaging and message framing on young smokers. Tobacco Control 2015;24:e87-e92.

36. Popova L, Owusu D, Jenson D, Neilands TB. Factual text and emotional pictures: overcoming a false dichotomy of cigarette warning labels [published online ahead of print, 2017 Apr 20]. Tob Control. 2017;tobaccocontrol-2016-053563. doi:10.1136/tobaccocontrol-2016-053563

37. Nan X, Iles I, Yang B, Ma Z. Public Health Messaging during the COVID-19 Pandemic and Beyond: Lessons from Communication Science. Health Communication. 2022;37(1):1-19. doi: 10.1080/10410236.2021.1994910

38. Lasswell, H. D. (1948). The structure and function of communication in society. In L. Bryson (Ed.), *The communication of ideas* (pp. 37–51). Harper and Row.

39. Kornfield R, Smith KC, Szczypka G, Vera L, Emery S. Earned media and public engagement with CDC's "Tips from Former Smokers" campaign: an analysis of online news and blog coverage. J Med Internet Res. 2015;17(1):e12. Published 2015 Jan 20. doi:10.2196/jmir.3645

For peer review only

40. Bennett P, Noble S, Johnston S, et al COVID-19 confessions: a qualitative exploration of healthcare workers experiences of working with COVID-19. BMJ

For peer review only

Figure 1 - Outline of higher-order and lower-themes on COVID-19 communication strategies

Uploaded separately as PDF as per editorial office request.

For peer review only

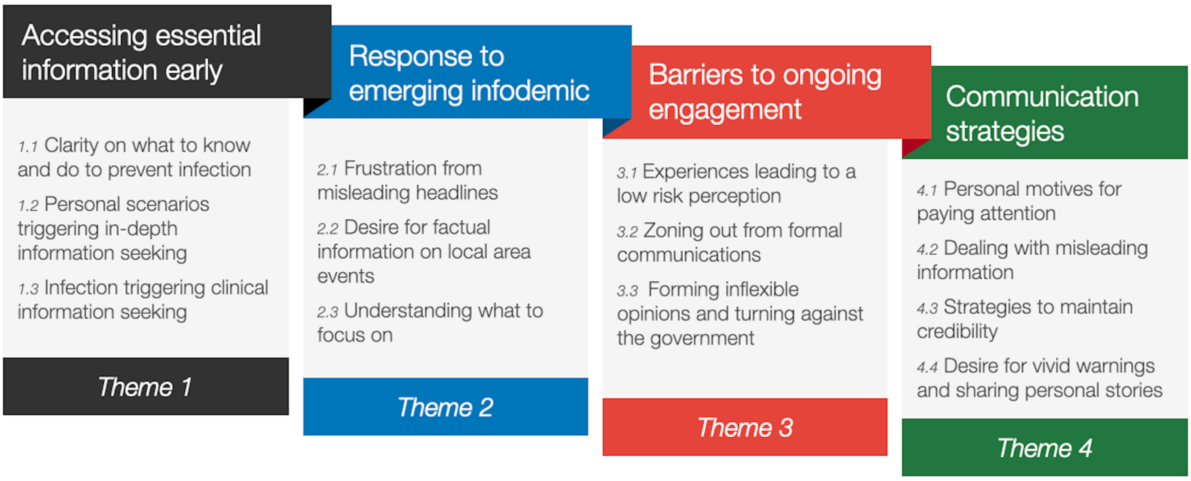

For peer review only

Standards for Reporting Qualitative Research (SRQR)*

http://www.equator-network.org/reporting-guidelines/srqr/

Page/line no(s).

Title and abstract

**Title** - Concise description of the nature and topic of the study Identifying the study as qualitative or indicating the approach (e.g., ethnography, grounded theory) or data collection methods (e.g., interview, focus group) is recommended	1
**Abstract** - Summary of key elements of the study using the abstract format of the intended publication; typically includes background, purpose, methods, results, and conclusions	3

Introduction

**Problem formulation** - Description and significance of the problem/phenomenon studied; review of relevant theory and empirical work; problem statement	5
**Purpose or research question** - Purpose of the study and specific objectives or questions	5

Methods

**Qualitative approach and research paradigm** - Qualitative approach (e.g., ethnography, grounded theory, case study, phenomenology, narrative research) and guiding theory if appropriate; identifying the research paradigm (e.g., postpositivist, constructivist/ interpretivist) is also recommended; rationale**	7
**Researcher characteristics and reflexivity** - Researchers' characteristics that may influence the research, including personal attributes, qualifications/experience, relationship with participants, assumptions, and/or presuppositions; potential or actual interaction between researchers' characteristics and the research questions, approach, methods, results, and/or transferability	8
**Context** - Setting/site and salient contextual factors; rationale**	6
**Sampling strategy** - How and why research participants, documents, or events were selected; criteria for deciding when no further sampling was necessary (e.g., sampling saturation); rationale**	6
**Ethical issues pertaining to human subjects** - Documentation of approval by an appropriate ethics review board and participant consent, or explanation for lack thereof; other confidentiality and data security issues	2
**Data collection methods** - Types of data collected; details of data collection procedures including (as appropriate) start and stop dates of data collection and analysis, iterative process, triangulation of sources/methods, and modification of procedures in response to evolving study findings; rationale**	5

For peer review only - http://bmjopen.bmj.com/site/about/guidelines.xhtml

**Data collection instruments and technologies** - Description of instruments (e.g., interview guides, questionnaires) and devices (e.g., audio recorders) used for data collection; if/how the instrument(s) changed over the course of the study	6
**Units of study** - Number and relevant characteristics of participants, documents, or events included in the study; level of participation (could be reported in results)	7
**Data processing** - Methods for processing data prior to and during analysis, including transcription, data entry, data management and security, verification of data integrity, data coding, and anonymization/de-identification of excerpts	6
**Data analysis** - Process by which inferences, themes, etc., were identified and developed, including the researchers involved in data analysis; usually references a specific paradigm or approach; rationale**	7
**Techniques to enhance trustworthiness** - Techniques to enhance trustworthiness and credibility of data analysis (e.g., member checking, audit trail, triangulation); rationale**	7

Results/findings

**Synthesis and interpretation** - Main findings (e.g., interpretations, inferences, and themes); might include development of a theory or model, or integration with prior research or theory	8
**Links to empirical data** - Evidence (e.g., quotes, field notes, text excerpts, photographs) to substantiate analytic findings	Table 1-4

Discussion

**Integration with prior work, implications, transferability, and contribution(s) to the field -** Short summary of main findings; explanation of how findings and conclusions connect to, support, elaborate on, or challenge conclusions of earlier scholarship; discussion of scope of application/generalizability; identification of unique contribution(s) to scholarship in a discipline or field	12
**Limitations** - Trustworthiness and limitations of findings	16

Other

**Conflicts of interest** - Potential sources of influence or perceived influence on study conduct and conclusions; how these were managed	2
**Funding** - Sources of funding and other support; role of funders in data collection, interpretation, and reporting	2

*The authors created the SRQR by searching the literature to identify guidelines, reporting standards, and critical appraisal criteria for qualitative research; reviewing the reference lists of retrieved sources; and contacting experts to gain feedback. The SRQR aims to improve the transparency of all aspects of qualitative research by providing clear standards for reporting qualitative research.

For peer review only

For peer review only - http://bmjopen.bmj.com/site/about/guidelines.xhtml

**The rationale should briefly discuss the justification for choosing that theory, approach, method, or technique rather than other options available, the assumptions and limitations implicit in those choices, and how those choices influence study conclusions and transferability. As appropriate, the rationale for several items might be discussed together.

Reference:

O'Brien BC, Harris IB, Beckman TJ, Reed DA, Cook DA. **Standards for reporting qualitative research: a synthesis of recommendations.** *Academic Medicine*, Vol. 89, No. 9 / Sept 2014
DOI: 10.1097/ACM.0000000000000388

For peer review only
